# Radiocarbon dating of lead white: novel application in the study of polychrome sculpture

**DOI:** 10.1038/s41598-021-91814-y

**Published:** 2021-06-24

**Authors:** Sara Sá, Laura Hendriks, Isabel Pombo Cardoso, Irka Hajdas

**Affiliations:** 1 Department of Conservation and Restoration and LAQV–REQUIMTE , NOVA School of Science and Technology, Caparica, Portugal; 2 Laboratory of Ion Beam Physics, ETH Zurich, Zurich, Switzerland; 3 Laboratory of Inorganic Chemistry, ETH Zurich, Zurich, Switzerland; 4 Institut Chemtech, School of Engineering and Architecture of Fribourg, HES-SO University of Applied Sciences and Arts Western Switzerland, Delémont, Switzerland

**Keywords:** Chemistry, Physics

## Abstract

Recently, radiocarbon dating underwent considerable technological advances allowing unprecedented sample size downscaling. These achievements introduced novel opportunities in dating cultural heritage objects. Within this pioneering research, the possibility of a direct ^14^C dating of lead white pigment and organic binder in paint samples was investigated on polychrome sculptures, a foremost artistic expression in human history. The polychromy, an indivisible part of polychrome sculpture, holds a key role in the interpretation and understanding of these artworks. Unlike in other painted artworks, the study of polychromies is repeatedly hampered by repaints and degradation. The omnipresence of lead white within the original polychromy was thus pursued as dating proxy. Thermal decomposition allowed bypassing geologic carbonate interferences caused by the object's support material, while an added solvent extraction successfully removed conservation products. This radiocarbon dating survey of the polychromy from 16 Portuguese medieval limestone sculptures confirmed that some were produced within the proposed chronologies while others were revised. Within this multidisciplinary study, the potential of radiocarbon dating as a complementary source of information about these complex paint systems guiding their interpretation is demonstrated. The challenges of this innovative approach are highlighted and improvements on sampling and sample preparation are discussed.

## Introduction

Radiocarbon dating has been increasingly used to aid in the study of artworks e.g.^1–3^. Today, state-of-the-art equipment enables the analysis of samples in the range of tens of micrograms of carbon^4–6^. With decreasing sample size the effect of contamination increases and thus requires monitoring and possible correction^7–9^.

As a result of the sample size reduction, new possibilities to apply radiocarbon dating in the field of cultural heritage have arisen. For example, targeting the pictorial layer on paintings is now feasible. The first milestone in this direction was the selective dating of the organic binder on paint layers^10^, followed by the possibility to date specific pigments, pushing the technique another leap forward. In particular, lead white, a synthetic inorganic pigment composed mainly of the lead carbonates hydrocerussite (2PbCO_3_·Pb(OH)_2_) and cerussite (PbCO_3_), incorporates the ^14^C signature of the atmosphere during its production by the traditional stack process. Thus the ^14^C dating of lead white can be used as dating proxy^11–13^, which is of great significance since this pigment was the main and essentially the unique white pigment used since Antiquity and is almost invariably present in European paintings and polychromies produced before the twentieth century^14^.

However, radiocarbon dating of lead white is challenging as the presence of other carbonates may interfere with the dating process, where the difficulty resides in the selective isolation of the lead carbonate's ^14^C signature. For instance, calcium carbonate (calcite) or calcium magnesium carbonate (dolomite), two fillers of geologic origin and commonly found in paint mixtures, are radiocarbon depleted, resulting in ages older than the production of lead white^13,15^. So far, two protocols have been proposed for the analysis of the lead carbonate: acid hydrolysis or thermal decomposition, both resulting in the decomposition of the carbonate and release of carbon dioxide. The choice of an appropriate approach highly depends on the purity of the lead white, i.e., the presence of other carbonate sources/contaminants^16^. Hydrolysis is effective for pure lead white material, while the thermal approach seems better suited for more complex samples, such as paint material, where an enhanced process selectivity is necessary^15–17^. Radiocarbon dating remains, however, an invasive technique requiring sampling. Thus, it is desirable to maximize the information that can be retrieved from a sole sample when granted. To this end, a reviewed proposal suggests the isolation of the lead carbonate through thermal separation, followed by exposure of the sample to hydrochloric acid to remove the carbonate from calcite and dolomite fillers, enabling further analysis of the organic binder^15^.

These revolutionary advances open the possibility for much vaster applications of radiocarbon dating on entirely new categories of artworks, namely on polychrome sculptures carved in limestone or marble. In contrast to other art objects, such as wooden sculptures and paintings on canvas, polychrome stone sculptures do not have an organic substrate to be analysed. Providentially, lead white was ubiquitous on the decoration of these sculptures. Therefore, the possibility of radiocarbon dating the polychromy materials is a major breakthrough for the study of these artworks.

Unlike other artistic painted surfaces, the polychrome finishing of the sculptures would be periodically renovated since the time of their creation supposedly to hinder the poor preservation state of the previous polychromy or to answer to changes of cultural taste and style^18^. Today, many polychrome sculptures present very intricate surfaces as a result of the consecutive reapplication of new layers of polychromy over the centuries, and of the degradation and loss of paint material, as well as uneven removal of repolychromy layers in past restoration interventions. Consequently, the understanding and interpretation of these surfaces and the identification of the original polychromy are very complex, demanding the assistance of different sources of information. If proven successful, the application of radiocarbon dating on paint samples from stone sculptures could serve as a powerful tool to assist in the study of these artworks' intricate polychrome surfaces – both original and repolychromies.

Herein, to test this possibility paint samples from 16 Portuguese medieval polychrome sculptures on limestone carved between the 14^th^ and 15^th^ centuries were selected for radiocarbon dating to confirm or dispel that the analysed polychromies were produced during the chronology under study. These artworks integrate a large group of polychrome sculptures that is being studied in a systematic and comparative manner where material and technical analyses are coupled with historical considerations, with the aim to shed light on the materials and techniques chosen for the original polychromy by its makers. Despite being an indivisible part of polychrome sculptures and a fundamental aspect for the understanding of this cultural expression, the polychromy of Portuguese medieval sculpture has been disregarded. The aim of this ongoing comparative material culture study, which is being undertaken in close collaboration with scholars from different disciplines, such as historians, geologists and biologists, is to contribute to the understanding of medieval polychromy by integrating the findings on Portuguese polychromies within a wider geographical and chronological context.

This paper tests the pioneering application of radiocarbon dating on paint samples from sculptures carved in limestone. Here, we explore this technique's viability and critically evaluate its integration in the complementary approach used to identify and study the complex paint stratigraphies found in stone sculptures.

## Materials and methods

### The object of study

The 16 objects selected for radiocarbon dating belong to two Portuguese national museums, Museu Nacional de Arte Antiga, in Lisbon, and Museu Nacional Machado de Castro, in Coimbra. The selected sculptures are attributed to the workshops of three leading masters: seven sculptures are attributed to Pero, a 14th century sculptor (P1 to P7), six to João Afonso (J1 to J6), whose activity is documented between 1439-40 and 1469, and three to Diogo Pires-o-Velho (D1 to D3), who is thought to have worked from the third quarter of the 15^th^ century to the mid-1510s (Table 1). The sculptures present several consecutive polychromy layers that have been applied over the centuries. However, the study focuses on the original/first polychromy of these artworks. All sculptures exhibit evidence of restoration interventions such as partial removal of repolychromy layers, consolidant materials or surface coatings. Prior to collecting so many samples on different objects, a preliminary study was conducted on sample P7 only, which upon conclusive results initiated the whole case study.

**Table 1:** List of sculptures selected for radiocarbon dating of their polychromy with respective workshop attribution and production date.

ID	Name	Inventory no.	Workshop	Date
P1	Virgin and Child	MNMC 3995	Master Pero	14th century
P2	Virgin and Child	MNMC 4069	Master Pero	14th century
P3	Expectation of the Blessed Virgin Mary	MNMC 645	Master Pero	14th century
P4	Saint Clare	MNAA 1077 Esc	Master Pero	14th century
P5	Virgin and Child	MNAA 984 Esc	Master Pero	14th century
P6	Expectation of the Blessed Virgin Mary	MNAA 1090 Esc	Master Pero	14th century
P7	Virgin and Child	MNAA 1087 Esc	Master Pero	14th century
J1	Altarpiece of Corpus Christi	MNMC 4023	Master João Afonso	15th century
J2	Virgin Mourning the Dead Christ	MNAA 1046 Esc	Master João Afonso	15th century
J3	Virgin and Child	MNAA 1076 Esc	Master João Afonso	15th century
J4	Our Lady of the Milk	MNAA 1042 Esc	Master João Afonso	15th century
J5	Saint Stephen	MNAA 1027 Esc	Master João Afonso	15th century
J6	Virgin and Child	MNAA 1001 Esc	Master João Afonso	15th century
D1	Saint Sebastian	MNAA 546 Esc	Master Diogo Pires-o-Velho	Third quarter of the 15th century to the mid-1510s
D2	Our Lady of the Rosary	MNAA 941 Esc	Master Diogo Pires-o-Velho	Third quarter of the 15th century to the mid-1510s
D3	Holy Trinity	MNAA 957 Esc	Master Diogo Pires-o-Velho	Third quarter of the 15th century to the mid-1510s

### Identification and study of the first polychromy

The study of the sculptures’ polychromies began with the thorough macroscopic and microscopic observation and documentation of the sculptures' surface. This was followed by selection, documentation, and removal of paint samples from the different polychrome areas of each sculpture. The collected paint samples were prepared as cross-sections and examined by optical microscopy (OM) using both visible light and ultraviolet radiation to identify the first polychromy and to understand its painting technique through the layer stratigraphy (see Supplementary Information: Instruments and conditions of analysis).

Identification of painting materials—pigments and fillers—of the first polychromy was performed using the complementary analytical techniques micro-Raman spectroscopy, micro-energy-dispersive X-ray fluorescence (µ-EDXRF) and scanning electron microscopy equipped with energy-dispersive X-ray spectroscopy (SEM–EDX) (for the material characterization of the samples see Supplementary Table S1). The study of binders was carried out with Fourier transform infrared spectroscopy in attenuated total reflection mode (FTIR-ATR) to confirm the natural origin of the binder. This characterization was undertaken at the Department of Conservation and Restoration at FCT-UNL.

As a result of this methodology, it was possible to characterise and to identify the first polychromy—the target of this study—from 13 out of the total of the 16 sculptures under study. In three sculptures attributed to master Pero (P3, P5 and P6) the identification of the first polychromy had not yet been possible due to their very complex and inconsistent surface. Thus, paint samples were collected in areas which correspond to what supposedly is the oldest polychromy with the hope to gain some understanding of the stratigraphy, potentially providing useful chronological information regarding possible previous polychromies. As this was a pioneering project, in some cases, a second sampling and additional steps in sample preparation were undertaken in an attempt to improve or to confirm the results. The collected microscopic and spectroscopic information forms the premise for the selection of samples for radiocarbon dating.

### Sampling criteria for radiocarbon dating

Areas for sampling for radiocarbon dating were selected according to material composition and accessibility of the intended layer on the sculpture. As mentioned above, the polychrome finishing of sculptures is not static, suffering many additions and alterations over time. This means that the targeted layer for radiocarbon dating, whether the first polychromy—as in the case of this work—or a repolychromy, is repeatedly disturbed and hidden under or intermingled with several other layers of polychromy, sometimes with a very similar appearance and composition. While similar layers can usually be distinguished during the study of the paint cross-sections, discrimination and physical separation during sample collection might be more troublesome and contamination of the targeted material with an undesired additional carbon source can occur. As far as external contamination to the targeted layer is concerned, restoration interventions are also an important factor to be accounted for. It is common knowledge that artworks are very often restored and the introduction of synthetic or natural consolidants and coatings on their surface might lead to erroneous analysis. Considering that many of these interventions are frequently not disclosed, not well documented, or not documented at all, the presence of such materials cannot be excluded based on the absence of information.

In the polychromies under study the existence of layers bearing lead white alone in the paint formulation is rare. In addition, in most cases, the paint layers are applied over a preparatory layer of complex composition, frequently involving several carbon-bearing pigments^19^. The separation of these two layers is virtually impossible owing to the strong cohesion between the two layers and their frequent thinness, which would result in a considerable loss of material if separation and isolation of the paint layer were attempted.

Also, the applicability of the radiocarbon dating of lead white method was initially questioned due to the limestone support, which could not be avoided when sampling. Indeed, a previous study concerning oil paint samples containing lead white and calcium carbonate reported a strong relationship between the curing state of the organic binder and the ^14^C age of lead white^15^. The introduction of a washing step was proposed to remove free fatty acid moieties originating from the oil binder, which would react with the variety of carbonates present. Here, since the paint matrix is already extensively hydrolysed, the presence of calcium carbonate was not expected to be a problem.

The presence of other carbon-bearing pigments is not expected to affect the lead white dating as the thermal preparation process is highly selective. Recent protocols allow for circumvent the interference of calcite and dolomite, however, the influence of other carbonates remains to be clarified. For instance, different decomposition temperatures have been proposed for azurite and malachite pigments^20,21^ with Frost and colleagues^21^ suggesting that decomposition of these carbonates starts below 350 °C. Thereby, selection of areas adjacent to paint layers including the geologic pigments azurite and malachite were avoided as their possible interference with the lead carbonate analysis due to similar decomposition temperatures are still to be investigated (but not included in the scope of this work).

Granted the opportunity, complementarily to lead white, the dating of the organic binder was also undertaken. However, in most cases, the lead white paint layers also included other carbon-bearing pigments such as carbon-based black, indigo, lac dyes, and calcium carbonate (see Supplementary Table S1). As mentioned above, sample treatment protocols involving acid hydrolysis enable the removal of carbonates from the paint formulations^10^. While old carbonates can be removed, the remaining carbon-bearing pigments will have an influence on the binder analysis as they are not removed. Henceforth when “binder” analysis is mentioned, it encompasses all carbon-bearing materials that remained for analysis. Among the man-made pigments in use during the medieval period, those made from a living organism (an organic source) carry the atmospheric ^14^C signal of their time of harvest/death. One can assume that such material was fresh, i.e., harvested and processed close to the time of use of the pigment, therefore has little offset to the age of the natural binding media. Since sample preparation for radiocarbon dating does not yet allow isolation and radiocarbon analysis of other datable pigments besides lead white, the analysis of paint samples that include such pigments will provide a mean radiocarbon measurement of all the carbon materials in the binder analysis. Problematic is the possible use of carbon black from mineral origin as the dead carbon signature would bias the result of the analysis, consequently, binder analysis should always be supported and compared to the lead white measurement.

In summary, considering the external sources of contamination and the complex composition of the polychromy material in these objects, the sampling process was established following the guidelines:

- Paint layers with a high lead white content were aimed for.
- Contaminations by conservation products were avoided whenever possible, but if unavoidable an adapted strategy to their removal was proposed in the next stage of the sample preparation.
- Areas including other carbonates such as azurite and malachite were avoided.
- Locations were selected according to the possibility to collect enough paint material, as well as where the separation of the intended layer from the most recent paint layers could be achieved.
- Two different areas corresponding to the same polychromy level were sampled in three of the sculptures to assess the compliance of the radiocarbon results and therefore the reliability of the established methodology.
- Samples were collected under a magnifying lens with a clean scalpel and kept inside a piece of aluminium foil for storage and transportation.

### Radiocarbon analysis

Samples were prepared following the most recent workflow as described in Hendriks, Kradolfer, et al. (2020)^22^, where both the lead white and binder ^14^C signature was gained following a two-step isolation procedure. Upon heating to 350 °C lead white thermally decomposes to lead oxide under the formation of CO_2_, which can be captured and measured. Other carbonates which decompose at higher temperatures were removed by acid hydrolysis allowing to date the carbon fraction of the binder. Although no conservation products were detected with FTIR-ATR analysis (spectral data was acquired on a PerkinElmer Frontier instrument in the Laboratory of Ion Beam Physics at ETH Zürich), whenever possible an additional step of solvent extraction was considered mandatory due to the typical restoration history of these artworks. This step was adapted from the solvent extraction procedure used to remove free reactive species originating from the natural binder which would react with the carbonates present^15^. As all washing steps lead to a certain degree of sample loss, powdery and small samples were directly thermally treated, whereas larger samples (> 2.5 mg) were washed. In addition, larger samples amounting to several milligrams (P1–P3) were split into two fractions where one was subjected to cleaning by acetone and ethanol (immersion for 12 h in each solvent at room temperature, then drying overnight at 60 °C) and the other was left untreated, hereby allowing an evaluation of the two approaches (see "Sample Preparation" column in Table 2). An exception was sample P7, the pilot sample. As the beneficial effect of additional solvent extraction was still under consideration at the time of the analysis, the sample was not washed despite its large size. Taking into account this evaluation, samples from the second sampling campaign were all washed (see “Viability of radiocarbon dating polychrome layers from stone sculptures” section). Details for the thermal preparation step may be found in Hendriks et al. (2020)^15^.

**Table 2:** ^14^C results of paint samples.

ID	ETH Lab code	Sample preparation	Targeted material	Weight (mg)	C mass (µg)	F^14^C ± 1σ	^14^C age ± 1σ (years BP)
P1	102536.1.1	Not washed	Carbonate	6.1	27	0.930 ± 0.008	585 ± 70
	102537.1.1	Not washed	Binder*		32	0.850 ± 0.007	1306 ± 69
	102536.2.1	Washed	Carbonate	2.6	18	0.896 ± 0.009	880 ± 78
	102537.2.1	Washed	Binder*		10	0.719 ± 0.012	2650 ± 129
P2	102538.1.1	Not washed	Carbonate	7.2	19	0.919 ± 0.010	682 ± 91
	102539.1.1	Not washed	Binder*		103	0.870 ± 0.007	1122 ± 63
	102538.2.1	Washed	Carbonate	6.2	14	0.919 ± 0.013	681 ± 114
	102539.2.1	Washed	Binder*		209	0.928 ± 0.007	603 ± 62
P3	102540.1.1	Not washed	Carbonate	1.1	8	0.958 ± 0.017	345 ± 145
	102541.1.1	Not washed	Binder*		15	0.851 ± 0.010	1299 ± 97
	102540.2.1	Washed	Carbonate	3.2	21	0.967 ± 0.009	273 ± 77
	102541.2.1	Washed	Binder*		75	0.951 ± 0.008	406 ± 64
P4	102568.1.1	Not washed	Carbonate	0.4	–	–	–
	102569.1.1	Not washed	Binder		–	–	–
P5	102552.1.1	Not washed	Carbonate	0.6	15	0.772 ± 0.009	2074 ± 97
	102553.1.1	Not washed	Binder*		7	0.967 ± 0.022	271 ± 182
	103832.1.1	Washed	Carbonate	1.3	8	0.883 ± 0.013	1000 ± 120
	103833.1.1	Washed	Binder*		18	0.953 ± 0.008	387 ± 64
P6	102558.1.1	Not washed	Carbonate	1.6	11	0.935 ± 0.019	540 ± 161
	102559.1.1	Not washed	Binder		4	1.000 ± 0.041	− 1 ± 333
	103834.1.1	Washed	Carbonate	1.3	10	0.969 ± 0.014	250 ± 114
	103835.1.1	Washed	Binder		0.5	1.091 ± 0.332	− 702 ± 2443
P7	88910.1.1	Not washed	Carbonate	4.6	68	0.925 ± 0.007	624 ± 58
	88910.2.1	Not washed	Carbonate	4.4	71	0.930 ± 0.006	581 ± 56
	88910.3.1	Not washed	Carbonate	2.1	24	0.911 ± 0.008	747 ± 73
	90421.1.1	Not washed	Binder*	1.5	171	0.920 ± 0.006	672 ± 54
	90421.2.1	Not washed	Binder*	2.0	189	0.922 ± 0.006	656 ± 53
	90421.3.1	Not washed	Binder*	0.6	79	0.929 ± 0.006	590 ± 53
J1	102542.1.1	Not washed	Carbonate	1.4	8	0.940 ± 0.017	501 ± 142
	102543.1.1	Not washed	Binder*		18	0.879 ± 0.009	1033 ± 85
J2a	102544.1.1	Not washed	Carbonate	2.4	22	0.945 ± 0.009	455 ± 75
	102545.1.1	Not washed	Binder		160	0.942 ± 0.008	482 ± 69
J2b	102546.1.1	Not washed	Carbonate	1.5	29	0.945 ± 0.008	457 ± 67
	102547.1.1	Not washed	Binder*		143	0.936 ± 0.008	527 ± 67
J3	102550.2.1	Washed	Carbonate	3.1	21	0.950 ± 0.008	415 ± 71
	102551.2.1	Washed	Binder		193	0.957 ± 0.007	352 ± 63
J4	103842.1.1	Washed	Binder*	0.3	3	0.942 ± 0.024	476 ± 205
J5a	102560.1.1	Not washed	Carbonate	0.4	6	1.011 ± 0.021	− 88 ± 166
	102561.1.1	Not washed	Binder*		3	1.287 ± 0.100	− 2029 ± 621
	103836.1.1	Washed	Carbonate	0.8	6	1.026 ± 0.016	− 206 ± 127
	103837.1.1	Washed	Binder*		5	1.246 ± 0.020	− 1766 ± 127
J5b	102562.1.1	Not washed	Carbonate	0.5	9	0.944 ± 0.020	460 ± 167
	102563.1.1	Not washed	Binder*		5	0.951 ± 0.032	407 ± 273
	103838.1.1	Washed	Carbonate	0.5	5	0.992 ± 0.029	66 ± 236
	103839.1.1	Washed	Binder*		2	0.885 ± 0.028	985 ± 257
J6	102564.1.1	Not washed	Carbonate	0.4	8	0.973 ± 0.017	222 ± 141
	102565.1.1	Not washed	Binder*		7	0.971 ± 0.023	232 ± 192
D1	102566.1.1	Not washed	Carbonate	0.6	9	0.970 ± 0.017	242 ± 139
	102567.1.1	Not washed	Binder		11	0.986 ± 0.016	115 ± 128
	103840.1.1	Washed	Carbonate	2.1	20	0.956 ± 0.008	357 ± 67
	103841.1.1	Washed	Binder		34	0.961 ± 0.007	317 ± 61
D2a	102554.2.1	Washed	Carbonate	2.5	25	0.950 ± 0.008	415 ± 68
	102555.2.1	Washed	Binder*		54	0.946 ± 0.008	443 ± 68
D2b	102557.2.1	Washed	Binder*	4.4	273	0.950 ± 0.008	416 ± 63
D3	102548.1.1	Not washed	Carbonate	2.2	27	0.953 ± 0.008	390 ± 66
	102549.1.1	Not washed	Binder*		134	0.918 ± 0.008	688 ± 67

The summary of results is organized by sample ID, ETH laboratory code, sample preparation (if washed with acetone and ethanol prior to the carbon isolation step), targeted material (lead carbonate or binder), the initial sample weight, the measured amount of carbon, the fraction modern (F^14^C) and the ^14^C ages with 1σ uncertainty. *Samples marked with a typographic asterisk contain an additional carbon source other than the binder, so the result should be interpreted with caution.

Radiocarbon measurements were performed on a MIni radioCArbon DAting System (MICADAS) instrument at the Laboratory of Ion Beam Physics at the ETH Zürich^23^, where the coupling of a gas interface allows the introduction of carbon dioxide from samples prepared in Pyrex tubes directly^5,24,25^ or following the combustion of samples in an elemental analyzer^6^. The F^14^C values of the samples were corrected for constant contamination^7,8^, then calibrated to calendar ages using the OxCal 4.4 software (https://c14.arch.ox.ac.uk/oxcal/OxCal.html)^26–28^ with the IntCal20 calibration curve^29^.

## Results and discussion

The following discussion is divided into two sections: the first addresses the feasibility of the approach, namely the sampling and sample preparation requirements, proposing improvements to the methodology, and the reliability of lead white paint from complex polychromy samples for dating these artworks. In the second section, the reported ^14^C ages are discussed in the broader context of understanding the polychromy, namely the assessment and interpretation of the calibrated age ranges obtained.

### Viability of radiocarbon dating polychrome layers from stone sculptures

The radiocarbon results are presented in Table 2 while an exhaustive material characterization of the paint samples can be found in Supplementary Table S1. The cross-reference between material analysis with ^14^C dating provided a more in-depth insight into the interpretation of the radiocarbon results.

In the context of the analyses of these artworks, two factors were challenging for the application of the radiocarbon dating technique: the first resided in the sample size compromise, and the second to avoid contamination of the sample.

Generally, samples of paint below 0.5 mg were too small to afford any meaningful results. Samples J4 and J5a, which weighed 0.3 and 0.4 mg respectively, yielded less than 10 µg C and provided a ^14^C date, while sample P4 with an initial weight of 0.4 mg proved to be too small to be measured at all (Table 2). It is to be observed that individual measurements of very small samples—with less than 1 mg of starting paint material and yielding less than 10 µg C—carried a significant error in the order of hundreds of years. The sample size thus plays a crucial role as the precision of the radiocarbon method largely depends on the counting statistics. Indeed, using AMS technology, the ^14^C atoms present in a sample are directly counted. While graphitized samples (0.2–1 mg of C) can provide high precision results down to 2‰ error^30,31^, gas targets are typically measured on less than 100 µg C with uncertainties ranging between 0.5 and 2% depending on the age of the sample^24,25,32,33^. A comparison of graphite and gas ^14^C age uncertainties conducted on foraminifera show that above 40 µg C age uncertainties increase as a function of increasing ^14^C age only, while below 40 µg C the decreasing sample size also plays a role^34^. This can be observed in the collected dataset as the samples are all assumed to have the same age but nonetheless show varying uncertainties, which increase for samples bearing less than 40 µg C. Not only is the sample size relevant with respect to the measured error, it dominates the reliability of the measurement; below tens of microgram C an indication may be gained, but the counting statistics are too low to be definite. For instance, the second sampling of P6 amounted to 1.3 mg, from which 10 µg C could be extracted from the lead white, which allows a reliable measurement, but less than half a microgram carbon was collected from the binder, resulting in an error of thousands of years.

Another contributing element to ^14^C age uncertainties is the constant contamination effect, which is also related to sample size and becomes increasingly significant as the sample size decreases. The typical reported laboratory contamination mass (m_c_) ranges between 0.3 and 1 μg C^9,35–37^ which, when considering a 1 mg sample represent less than 0.1%, but for a sample of 30 μg rises to a few percent. Naturally, the age of the contamination will affect how strong the age bias is. Generally, the m_c_ value shows the biggest variation between different laboratories while the fraction modern contamination (F^14^C_c_) value is fairly constant. Each measurement series were thus evaluated for constant contamination, where the calculated models indicated values ranging between F^14^C_c_ = 0.4–0.7 and m_c_ = 0.2–0.9 μg C between the different sequences. The model parameters reflect the long-term contamination values of the laboratory^8,9^ and through error propagation also contribute to the overall ^14^C age uncertainty. Generally, contaminants which are similar in age with the investigated sample, have a lower effect. Here, 30 μg C samples see their uncertainties increase in the order of 5–10 years, 20 μg C by 20 years, while sizes smaller than 10 μg C increase by more than 50 years. These increases can be considered as moderate in contrast to paleoclimate reconstructions studies dealing with foraminiferal samples spanning the last 20000 years, where this effect is particularly pronounced as the investigated material is both smaller and older^34^.

Sampling actions must be balanced between the necessary amount and generated damage, where moderately larger samples do not always achieve a considerable increase in precision. The sample's C content is not directly proportional to the initial sample weight since it depends on the sample composition. For instance, lead white contains less than 5% C, with the content varying according to the proportions of hydrocerussite and cerussite present in the pigment, meaning that a significantly larger sample is required to extract a minute amount of carbon as the pigment is further mixed with other compounds in the paint sample. Also, sample inhomogeneity must be considered (see sample P2 where upon similar initial starting weights, twice as much carbon was gained from the binder analysis in the second treatment). Nonetheless, from this study, for the carbonate analysis, samples weighing around 2 mg (such as D2a and D3) contained sufficient lead white, yielding meaningful results.

The lead white’s radiocarbon ages of the collected samples are in the range of hundreds of years, which is within the expected values for the medieval period. Overall, no interferences from the limestone support were observed in the ^14^C signature of the lead white pigment. Only two measurements stand out, the washed fraction of P1 and P5. The divergence observed in P1 is subtle, as the non-washed fraction is in line with expectations (585±70 years BP, resulting in the calibrated age range 1284–1438 AD). On the contrary, the washed sample revealed a too old age (880 ± 78 years BP, calibrated age range 1025-1275 AD). These discrepant results are puzzling and allow postulating different hypothesis while highlighting the issue of sample inhomogeneity. The presence of conservation materials that were not initially detected and thus not removed in the solvent extraction step is inferred to be the source of the error. Indeed, in the event that conservation materials were unevenly present in the sample and had a decomposition temperature inferior or similar to the one of lead white, a bias in the lead carbonate measurement could be observed. Following this hypothesis, samples from the same sampling area were later re-examined. Under the microscope, a thin transparent film was observed over some of the paint fragments. FTIR-ATR analysis to the surface of these fragments revealed a close match with polyvinyl acetate (PVAc) (see Supplementary Fig. S1). In contrast, within the same sample, the fragments that did not exhibit this transparent film on the surface featured the typical infrared bands belonging to lead white in an oil-based binding media. The results outcome indicate a poor choice of sample treatment, while the approach in washing the larger aggregates, which were hold together by the consolidant, was well thought, the choice of solvent was inadequate. PVAc derived materials have proved to be hard to remove in previous studies where different organic solvent associations were pursued^38^. Furthermore, above 285°C PVAc starts to degrade under elimination of acetic acid, then with increasing temperature the polymer chain is reported to further break down by chain scission reactions^39,40^. Fortunately, the unwashed fraction, which consisted of sparse smaller fragments was free of the PVAc consolidant and thus explains the observed age difference. Further evidence of the presence of restoration products in the different objects was demonstrated in the binder's analysis—see the following section). Thus, it is important to identify such compounds in order to adjust the choice of solvent and favour their removal, and henceforth ensure the selectivity of the process.

Another point worth mentioning is the potential impact of lead white degradation products. Laurionite and plumbonacrite, identified in the paint layer, have been associated with this phenomena in the literature, although the mechanisms of degradation are not yet fully understood^41–43^. Therefore, this subject deserves a more in-depth study to evaluate if this could be a cause of discrepancy in ^14^C results.

Sample P5 also stands out by its considerably old carbonate measurements that contrast with the age gained from the organic binder material. While the use of a mineral source of lead white is a possibility, it is considered to be very unlikely. Other possible sources of contamination could be proposed, such as contamination with copper carbonate residues linked to a nearby azurite repolychromy or with a conservation material, as proposed for sample P1.

Nonetheless, within the collected dataset the lead white ^14^C ages generally show little deviation between washed and not washed samples. These results tend to indicate that the thermal treatment strategy has the advantage of being material-specific, since only the inorganic lead carbonate reacts to carbon dioxide independently of any other carbon-containing material.

In contrast, in what concerns the binder dating, the proposed additional washing step seemed to have a much deeper effect. Indeed, the ^14^C ages collected from the binder show a much higher degree of scatter. While many fall within the anticipated range (P5, P7, J2a/b, J3, J4, J5b, J6, D1, D2a/b), others show strong deviations. As predicted, some of the samples which did not undergo a solvent extraction step prior the thermal combustion are typically several thousand years older in comparison to their duplicate which was washed. This is particularly obvious in the split samples P2 and P3 (see Table 2). This indicates that an additional carbon source material carrying a depleted ^14^C signal, such as conservation products, was added to the object but removed from the samples through the solvent extraction step. The agreement between the ^14^C ages of the washed binder material with the carbonate reinforces this hypothesis. Samples J1 and D3 again support this hypothesis as they were not subjected to this additional washing step, as the risk of the powdery sample loss dominated in the decision making and the recovered ^14^C age of the binder is also biased. Although FTIR-ATR was performed in some of the paint fragments to evaluate the presence of restoration products, none were detected. An explanation could be linked to the small sample size, and/or to the presence of consolidants below the detection limit, their uneven distribution on the samples, or being hidden by overlapping spectral bands. In order to achieve better control of the sample, it is proposed for future studies the examination of the sample fragments collected for radiocarbon dating under the microscope, under normal light and ultraviolet radiation. At the same time, the indicative presence of a conservation product must be identified through complementary analytical techniques.

Results from samples J5b and P1 are particular as the binder's washed fraction is twice as old as the non-washed fraction, hereby questioning our explanation. The small size and considerable error of sample J5b is most definitely the cause, where the washed fraction yielded only 2 µg C, resulting in low C currents and poor counting statistics. On the other hand, sample P1 was larger, therefore, sample size is not an argument. The unsuccessful removal of PVAc, a commonly used conservation product which has been proved difficult to remove when aged, and that was unevenly present in the sample, is most certainly the explanation to the older ages observed for the washed fraction as it is also the reason for the age bias on the lead white fraction. While extraneous carbon contaminants brought through conservation material are a distinct problem causing significant age deviations, the presence of carbon matter other than the binder within the sample is not so clear. The too old results observed on the non-washed fraction whose carbonate counterpart is thought free of PVAc may be caused by sample inhomogeneity and composition. Indeed, within the corresponding paint layer, particles of carbon black have been identified (see Supplementary Table S1). Carbon-based black pigments may have various origins from burning organic matter to a mineral origin. Thus, in the event that older organic matter was burnt to produce the pigment, or if a mineral (^14^C depleted) source of carbon black was used, such deviation in the measured binder's age could be observed. While carbon black particles were also found in other samples with seemingly no interferences, its presence must be addressed with caution; it is difficult to assess the exact effect of carbon black particles as this depends on their source, their concentration and the inhomogeneity of the paint composition. This emphasizes the importance of the sample’s characterization.

An important point to highlight is the variability of the group of results. While the problem of sample inhomogeneity is inferred in some cases, samples P7 and J2, which were split in replicates, are in excellent agreement among and between the targeted materials. The replicates can then be averaged to increase the precision of the estimate, where the sample inhomogeneity is comprised in the experimental variability (noise).

In summary, the presence of conservation products has a large and undesired impact on the ^14^C dating results. In most cases, the problem seems to be solved through the solvent extraction step. The fact that restoration interventions are not always documented reinforces the mandatory status of this step. Nonetheless, it must be considered that some sample loss will occur. This problem can be greatly minimised by collecting fragments instead of scraping of the surface, hereby avoiding powdery samples. This facilitates sample handling and largely prevents sample loss. As the ideal sample is not always possible, improvements on the solvent extraction step and solvent choice, as well as the identification of restoration products, and possible adapted solvent choice need to be addressed in future research to allow more reliable dating of the binder. Moreover, owing to the ineluctable presence of additional carbon source within the organic binder, its dating results from an average of the different components and its interpretation should always be supported and compared to the lead white measurement. On the question of lead white as ^14^C clock, it is encouraging to observe that the results do not seem to be affected by the presence of most of the synthetic conservation products, therefore stressing the selectivity and robustness of the thermal approach, and overall, the potential of the method.

### Radiocarbon ages in the context of understanding polychromy

The 16 artworks under study, like other polychrome stone sculptures, represent very challenging case studies. The ensemble of the current knowledge about each of these sculptures, such as the probable production dates resulting from the attribution to a specific master sculptor^e.g.^^44^ and the belief that the polychromy would be performed near the date of the carving^45^—both according to previous historical studies—as well as the crucial study and identification of the first polychromy, set the foundation for the interpretation of radiocarbon dating results. In terms of the selection of paint samples for sampling, these objects could be considered the worst-case scenario. For this reason, and due to the multiple possible causes of interference during the analyses, the measurement of such complex paint samples should always be performed complementarily to the material and technical study of the polychromy and the historical study of the artwork. Results should always be interpreted carefully, and the conclusions drawn should be cautious.

Generally, when dealing with artworks, the radiocarbon measurement of paint samples is hampered by the small sample size requirements, resulting in relatively large uncertainties of the reported ^14^C ages. The respective measurement uncertainty hereby plays an important role in the calibration step, where ^14^C ages are converted to calendar ages. The latter may be narrower or broader depending on which part of the calibration curve is intersected. In the case of the medieval period, a precise calibration to real calendar ages is hindered by ^14^C fluctuations in the calibration curve^46,47^. As a result, individual measurements of very small samples were generally too broad to provide a meaningful interpretation, as illustrated by samples J1 and J4.

**Figure 1:**
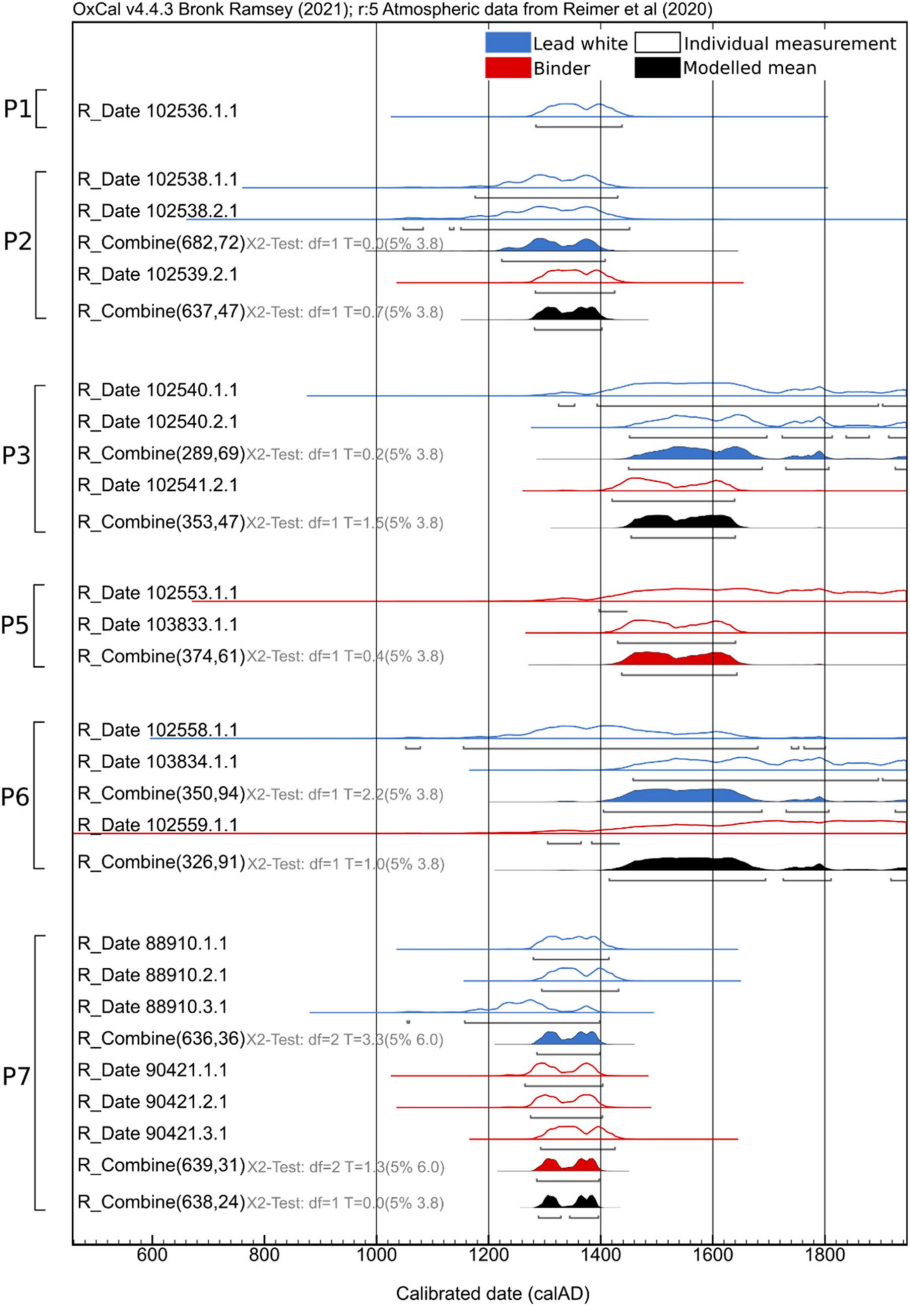
Calibrated ^14^C ages for the samples of Pero’s workshop. The simple radiocarbon calibration of the ^14^C ages of lead white (blue) and binder (red) are displayed as outlines while the solid distributions represent their mean value using the combine function in Oxcal.

In the event of multiple radiocarbon measurements, it is justifiable to calculate a weighted mean with an associated error to improve the precision and hereby narrow the possible calendar ages window. Ward and Wilson^48^ were the first to detail the combination of individuals dates using a statistical method, which has now become a standard practice for comparing and combining a series of ^14^C dates in the literature. Within this approach, a chi-square test is automatically performed and allows assessing whether the association between the variables is statistically significant, i.e., how good the ^14^C dates agree among each other, a useful tool in archaeological context for comparing whether a set of samples have the same origin as demonstrated by Edinborough et al.^49^. In the present dataset, two individual measurements were gained from a same sample by specifically targeting first the lead white carbonate, then the natural organic binder. The two materials are assumed to belong to the same time period, where the event of interest is the painting act. Using the R_Combine() function in Oxcal both the lead white’s and binder’s respective ^14^C age were pooled, where the final combined results considerably narrow the possible time window of creation of the polychromies under study (which is further discussed within the different workshops below). The consistency of the combined results of the uncalibrated measurements is put forward by the chi-square test which is compared with a chi-square distribution. Generally, the value is smaller which indicates a conservative tendency (see Fig. 1 and Supplementary Fig. S2 and S3 for the OxCal R_Combine date result plots for each group of measurements detailed on Table 3).

**Table 3:** Calibrated age ranges of the paint samples.

ID	Targeted material	^14^C age ± 1σ (years BP)	Calibrated age range (AD) (95.4% confidence interval)	Combined ^14^C age ± 1σ (years BP)	Combined age range (AD) (95.4% confidence interval)
P1	Carbonate	585 ± 70	1284–1438	–	–
P2	Carbonate	682 ± 91	1176–1430	637 ± 47	1282–1402
	Carbonate	681 ± 114	1048–1083 & 1130–1138 & 1150–1451		
	Binder	603 ± 62	1283–1425		
P3	Carbonate	345 ± 145	1325–1353 & 1393–1895 & 1903–1950	353 ± 47	1454–1640
	Carbonate	273 ± 77	1451–1696 & 1724–1813 & 1838–1879&1914–1950		
	Binder	406 ± 64	1420–1639		
P5	Binder	271 ± 182	1397–1950	374 ± 61	1437–1643
	Binder	387 ± 64	1430–1640		
P6	Carbonate	540 ± 161	1052–1078 & 1155–1680 & 1740–1753 & 1763–1800	326 ± 91	1415–1694 & 1726–1811 & 1918–1950
	Binder	− 1 ± 333	1305–1365 & 1384–1950		
	Carbonate	250 ± 114	1458–1895 & 1903–1950		
P7	Carbonate	624 ± 58	1280–1414	638 ± 24	1289–1329 & 1345–1396
	Carbonate	581 ± 56	1295–1432		
	Carbonate	747 ± 73	1055–1058 & 1157–1398		
	Binder	672 ± 54	1265–1403		
	Binder	656 ± 53	1275–1403		
	Binder	590 ± 53	1293–1425		
J1	Carbonate	501 ± 142	1219–1675 & 1743–1750 & 1765–1799	–	–
J2a	Carbonate	455 ± 75	1321–1358 & 1390–1637	470 ± 51	1324–1355 & 1392–1515 & 1590–1620
	Binder	482 ± 69	1304–1367 & 1382–1524 & 1572–1630		
J2b	Carbonate	457 ± 67	1322–1357 & 1391–1529 & 1545–1635	492 ± 48	1320–1359 & 1389–1485
	Binder	527 ± 67	1290–1480		
J3	Carbonate	415 ± 71	1407–1641	380 ± 48	1442–1531 & 1538–1636
	Binder	352 ± 63	1440–1655		
J4	Binder	476 ± 205	1178–1192 & 1203–1950	–	–
J5b	Carbonate	460 ± 167	1226–1696 & 1724–1813 & 1838–1878 & 1915–1950	478 ± 111	1288–1644
	Binder	407 ± 273	1050–1080 & 1153–1950		
	Carbonate	66 ± 236	1442–1950		
	Binder	985 ± 257	564–1433		
J6	Carbonate	222 ± 141	1466–1950	226 ± 114	1483–1950
	Binder	232 ± 192	1411–1950		
D1	Carbonate	242 ± 139	1459–1950	306 ± 42	1475–1660
	Binder	115 ± 128	1515–1591 & 1620–1950		
	Carbonate	357 ± 67	1436–1657		
	Binder	317 ± 61	1446–1669 & 1781–1798		
D2a	Carbonate	415 ± 68	1410–1638	429 ± 49	1410–1525 & 1559 & 1631
	Binder	443 ± 68	1328–1345 & 1395–1637		
D2b	Binder	416 ± 63	1412–1532 & 1537–1636	–	–
D3	Carbonate	390 ± 66	1426–1641	–	–

The summary of results is organized by sample ID, targeted material, the ^14^C ages with 1σ uncertainty and the respective calendar ages calibrated using the software Oxcal 4.4.^26,27^ with the IntCal20 calibration curve^29^. The last two columns represent the ^14^C ages with 1σ uncertainty and the respective calendar ages generated by the combination of the individual results, which is checked for internal consistency by a chi-square test which is performed automatically by Oxcal.

Hereafter, two cases are considered (a schematic of which is to be found in Supplementary Fig. S4). Case A is where all measurements are made on the same sample, i.e., a single sampling site that provided sufficient material for multiple measurements. Here we assume a homogenous sample and combine the measurements with respect to material first, then all together, where the chi-square test allows dismissing any measurement or preparation error. Case B is for different samples, when two different sampling sites of the same polychromy phase are considered (see Supplementary Fig. S4). Indeed, owing to the superimposed repaints it is often difficult to ascertain sampling the same polychromy phase in different location. Thus, first the lead white and binder results are combined per sampling area, then the combination of the different sites is considered depending on whether the chi-square test is passed or not. In this manner, the paint stratigraphy interpretation which led to the sampling of the replicates is validated, while also providing a safeguard in case of an error in the material analyses or any contamination issue.

From the 16 investigated sculptures, 11 could indeed correspond to the original polychromy. Within the remaining sculptures, one was too small to be measured (P4), in three, the radiocarbon results confirmed that the analysed polychromies were not contemporary of the carving (P3, P5 and P6), and finally, for sculpture J5 results were inconsistent between the two sampled areas.

Among the objects attributed to the workshop production of master Pero, the polychromies of the sculptures P1, P2 and P7 carry the older ages and fit within the expected 14th century attribution (Fig. 1). Sample P7 shows the potential of ^14^C dating; this sample was split into three replicates and results are in good agreement among and between both targeted materials. Furthermore, their respective combination greatly increases the precision of the estimate. In contrast, the radiocarbon results confirmed that the polychromies of the sculptures P3, P5 and P6 were not contemporary of the carving. In fact, in all these three sculptures the paint stratigraphy showed inconsistencies that were already raising serious questions regarding the possibility of the identified polychromy being the original one.

**Figure 2:**
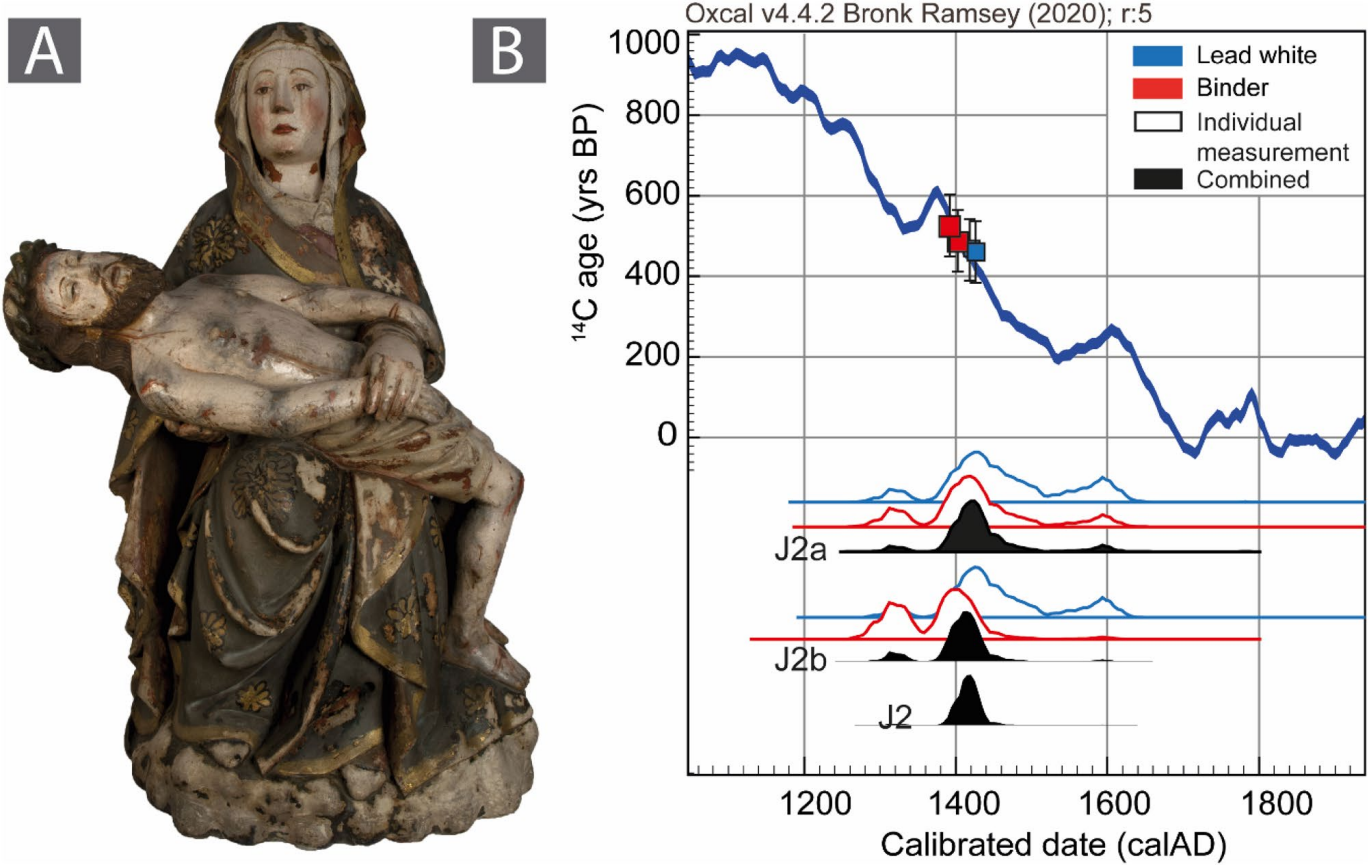
A) Portuguese medieval sculpture J2, attributed to the workshop of master João Afonso; B) The simple radiocarbon calibration of the ^14^C ages of lead white (blue) and binder (red) are displayed as outlines while the solid distribution (black) represents their mean value using the combine function in Oxcal. Following case B, the mean value of both J2a and J2b sampling sites were further combined (X^2^-test: df = 1 T = 0.1 (5% = 3.8)), where the chi-square test confirms that this association is statistically relevant. Alternate combination sequences are further discussed and compared in the figure S5 of the Supplementary Information.

The group of sculptures attributed to Master João Afonso were produced during the 15th century. The object J2, which was sampled at two different locations, showcases the potential of the method. First, a near to perfect agreement is observed between the ^14^C ages of the different targeted materials of samples J2a and J2b. The data were combined following case B, yielding two similar time windows beginning as early as the 1310s. Under the assumption that both sampling sites J2a and J2b belong to the same polychromy phase, the results were further pooled. The final result allows confirming that despite the difficulty of sampling, the same polychromy phase was sampled in both locations (validated by chi-square test) and the calibration interval is narrowed between 1399 and 1462 (mean ^14^C age of 482 ± 35 years) and thus confirming the age of the polychromy as 15^th^ century (Fig. 2).

Unfortunately, such precise attribution could not be confirmed in the other samples of this group. Results from sculptures J3 and J6 also indicate mid-15th as the earliest possible date of creation, although they do not exclude a later execution either, as the produced time window extend up to the 17th century and 1950, respectively. Samples J1 and J4 were simply too small (Table 2), bearing large uncertainty and as a consequence broad time windows. Interestingly, using the former IntCal13 calibration curve, both samples cover the 13th to 1950, while with the new calibration curve IntCal20, only J4 extends into the mid-20th while J1 goes as far as the 18th century. The analyses of these sculptures will be repeated, employing the proposed improved methodology to narrow the calibrated time ranges.

As for the sculpture J5, which was sampled in two different locations, different results were obtained for the two sampled areas (J5a and J5b). The two sampled paint layers have been interpreted during the material and technical study of the polychromy as belonging to the same phase of polychromy (the original), but the radiocarbon results indicate a much younger date for one of the sampled paint areas (J5a), where negative ^14^C ages are indicative of modern material post-1950. Nonetheless, owing to the minim sample size (< 10 µg C), the corresponding error could tilt the value into positive ages and thus over interpretation is a risk. Although, in the area where the sample carrying unexpectedly younger age was collected, the presence of younger paint layers with very similar appearance was observed and thus a plausible contamination should not be ruled out. A possible incorrect interpretation of the paint stratigraphy is not excluded and the polychromy will be further evaluated with this result in mind. Thus, despite having two replicates (case B), the dates from the two sampling sites were not further combined. Considering only the calibrated ages from sample J5b, an attribution to the 15th century is possible although a later attribution belonging to the mid-17th is not to be dismissed either.

Finally, the last set of measurements belonging to sculptures of the workshop of Diogo Pires-o-Velho, whose time of activity spread from the last quarter of the 15th century to the beginning of the 16th, are largely in agreement with this attribution. Despite all the calibrated time intervals expand into the mid-17th, the earliest date of creation begins in the 15th century, thus covering Diogo Pires-o-Velho period of activity. Owing to the particular shape of the calibration curve at this time period, i.e., a plateau, removing more material from the original layer will not suffice to narrow the obtained calibrated dates. A possible alternative solution would be to make use of stratigraphic information into the interpretation of the radiocarbon data by further sampling. A sequence could be defined based on the successive paint layers, which are known to follow one after another. The overlying could not be older than the one afore and thus gain in dating precision of the original and following polychromies. This strategy can be compared to archaeological or geological stratigraphic layers, for which absolute dates are obtained from the organic remains embedded within the different layers.

Overall, with the help of statistical method, multiple measurements may be combined affording narrower time windows, where regardless of the combination strategy the results do not vary when dealing with the same event (see Supplementary Fig. S5). In summary, while the radiocarbon dates cannot confirm the attribution of the polychromy to a given individual, they provide additional support to the art technological analysis confirming that the polychromies are within the expected time periods. In fact, in the frame of this study, radiocarbon dating assumes special importance as results attained regarding polychrome materials and techniques are somehow surprising. Namely, the observation that within the set of Portuguese polychrome sculptures a systematic use of coloured preparation layers strongly contrasts with the more common use of white layers reported in different studies regarding European polychrome sculptures; the use of different pigments to construct colour; and finally the use of different colour codes in the sculptures are challenging established paradigms^19^.

## Conclusion

The recent advances in radiocarbon dating have opened new opportunities for studying artworks. Within this study, the possibility of dating polychromies via the natural organic binder or lead white pigment without interference from the limestone support was demonstrated, and henceforth could be extended to other stone substrates such as marble. This achievement is a major breakthrough in the field of artwork analysis and hereby sets the first milestone in ^14^C dating of polychrome sculptures. Radiocarbon dating of the sculptures' polychromies confirmed that some of the polychromies were produced within the chronologies under study and helped to discriminate the ones that were not original. Within the multidisciplinary approach used to study sculptures' polychromy, the technique proved its potential in guiding the interpretation of the paint stratigraphies. Moreover, in the particular case of polychrome stone sculptures, the application of radiocarbon dating gains remarkable importance as the findings of this transdisciplinary study are defying art history's long-standing paradigms.

The paint samples with complex composition and collected from intricate and restored surfaces provided an opportunity to expose potential problems of analysing paint samples. The study enabled to identify the most critical steps of sample collection and preparation, highlighting challenges in sampling and the necessity of control via the replication of measurements. Moreover, it indicated the future direction of research regarding the effect of conservation materials and their respective removal. Most importantly, this study demonstrates that it is fundamental to integrate ^14^C results within a broader framework, such as the historical background of the artwork and the material and technical analysis of the paint stratigraphy in order to avoid misinterpretation of results.

## Supplementary Information

Supplementary Information.
