# Supplementary material for: Radiocarbon dating of lead white: novel application in the study of polychrome sculpture
Source: Sci Rep. 2021 Jun 24;11:13210. doi: 10.1038/s41598-021-91814-y (PMC8225632; doi:10.1038/s41598-021-91814-y)
Supplement: Supplementary file 1 — Supplementary Information. [file 41598_2021_91814_MOESM1_ESM.docx]

**Radiocarbon dating of lead white: novel application in the study of polychrome sculpture**

Sara Sá^1^, Laura Hendriks^2,3,4^, Isabel Pombo Cardoso*^1^, Irka Hajdas^2^

^1^Department of Conservation and Restoration and LAQV–REQUIMTE, NOVA School of Science and Technology, Caparica, Portugal

^2^Laboratory of Ion Beam Physics, ETH Zurich, Switzerland

^3^Laboratory of Inorganic Chemistry, ETH Zurich, Switzerland

^4^Institut Chemtech, School of Engineering and Architecture of Fribourg, HES-SO University of Applied Sciences and Arts Western Switzerland

*Corresponding author. Email: [isabel.pombocardoso@gmail.com](mailto:isabel.pombocardoso@gmail.com)

**Supplementary Information**

**Instruments and conditions of analysis**

Optical microscopy

The optical microscope used is an Axioplan 2ie ZEISS microscope equipped with incident halogen light illuminator (tungsten-halogen light source, HAL 100); UV radiation illuminator (mercury illuminator source, HBO 100); and a digital Nikon camera DXM1200F, with control software Nikon ACT-1 (version 2.62) (https://www.nikon.com/products/microscope-solutions/support/download/software/camerasfor/act1_v263.htm). Samples were analysed with 10x ocular lenses and 5x/10x/20x/50x objective Epiplan lenses (giving a total optical magnification of 50x, 100x, 200x, and 500x).

The samples were analysed under crossed polars – polariser and analyser filters; and for UV radiation the ZEISS filter set 02 [G 365, FT 395, LP 420] was used. The scales for all objectives were calibrated within the Nikon ACT-1 software.

µ-EDXRF

X-ray fluorescence spectra were obtained using an ArtTAX Pro spectrometer from Intax GmbH. Operating with a 30W molybdenum (Mo) X-ray tube, focusing polycapillary lens and silicon drift electrothermally cooled detector with a resolution of 160 eV at 5.9 keV (Mn-Kα). The accurate positioning system and polycapillary optics enable a small area of primary radiation (∅ ~70 μm) at the sample’s surface. Elemental compositions were obtained from the average of three independent spots, analysed with a tube voltage of 40kV and a current intensity of 300 or 600µA and live time 100s.

SEM-EDS

Samples were covered with a thin layer of gold and palladium under vacuum using the equipment Q150T ES, Quorum Technologies, to ensure the sample conductivity. SEM micrographs and elementary analysis were obtained using a scanning electron microscope (SEM) Hitachi S-2400, with a resolution of 4 nm (25kV, WD = 5 mm), acceleration voltage of 0.3 - 25kV and magnification of x20 – x300.000, coupled to a Quantax 2.0 Bruker detector. Micrographs were acquired using secondary (SE) and backscattered (BSE) electron detectors. SEM-EDS analysis were performed at MicroLab - Electron Microscopy Laboratory, Instituto Superior Técnico of the University of Lisbon.

FTIR-ATR

FTIR-ATR analyses performed at the Laboratory of Ion Beam Physics at the ETH Zürich were carried out in a PerkinElmer Frontier instrument (PerkinElmer Massachusetts, USA). Spectra were acquired in the 4000-550 cm^-1^ spectral region, with a resolution setting of 4 cm^-1^ and 8 scans.

FTIR-ATR analysis performed at the Department of Conservation and Restoration at FCT-UNL were carried out in an Agilent Handheld 4300 FTIR spectrophotometer equipped with a wire-wound source, ZnSe beam splitter, a Michelson interferometer and DTGS detector. All spectra were acquired with a diamond ATR module, in the 4000-650 cm^-1^ spectral region, with a resolution setting of 8 cm^-1^ and 64 scans.

µ-Raman

Micro-Raman microscopy was carried out using a LabRAM 300 HORIBA Jobin Yvon spectrometer, equipped with a He-Ne laser of 17 mW power operating at 632.8 nm and an external laser of 50mW power operating at 532 nm. Spectra were recorded as an extended scan. The laser beam was focused with Olympus objective lens 50x and 100x. The laser power at the surface of the samples was varied with the aid of a set of neutral density filters (optical densities 0.3, 0.6, 1).

**Material identification of the paint layers selected for radiocarbon dating**

Table S1. Sampled areas on the sculptures and material composition of the paint collected for radiocarbon dating. The summary of results is organized by identification number of the sculpture, description of sampled area, layers sampled for radiocarbon dating, and materials identified in these layers. **Bold**: pigments in higher concentration in the paint layer; [in square brackets]: pigments in very low concentration in the paint composition (qualitative evaluation); The numbers in the paint stratigraphy identify the layers sampled according to the following code: 0 indicates the stone substrate; 1 stands for the preparatory system (1A for the identified isolation layer, 1B and 1C for preparatory layers); numbers 2 and 3 stand for paint layers. Data leading to the identification of the binding media composition is not reported below but points to a natural source, oil and/or protein based.

**1A**

| ID | Sampling area | Corresponding stratigraphy | Analytical data | | Materials identified in the layers sampled for radiocarbon dating |
| --- | --- | --- | --- | --- | --- |
|  |  |  | General stratigraphy data | Layer specific data |  |
| P1 | Virgin's mantle | 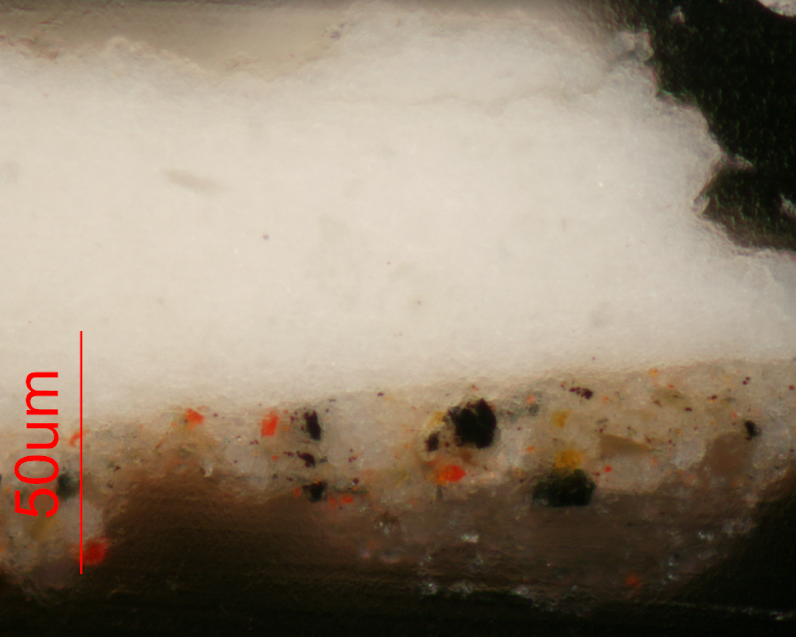  **0**  **1B**  **2** | EDXRF: **Ca**, **Pb**, Hg, Fe | **2. Raman:**  104 (s), 1051 (vs)  104 (s), 128 (s), 329 (w), 415 (m), 1050 (vs) | **Lead white (2PbCO_3_.Pb(OH)_2_ / PbCO_3_)**  Plumbonacrite (Pb_10_(CO_3_)_6_O(OH)_6_)? |
|  |  |  |  | **1B. Raman:**  104 (vs), 1050 (vs)  252 (vs), 282 (w, sh), 342 (m)  1325 (vs, br), 1595 (vs, br)  1008 (vs)  299 (m), 395 (vs)  107 (vs), 121 (s, sh), 273 (w), 327 (m)  **SEM-EDS**  Pb, O, Si, Al, Ca, Na, K, Mg | Lead white (2PbCO_3_.Pb(OH)_2_ / PbCO_3_)  Vermilion (HgS)  Carbon black (C)  [Calcium sulphate dihydrate (CaSO_4_.2H_2_O)]  [Goethite (α-FeOOH)]  Laurionite Pb(OH)Cl?  [Lake pigment]? |
| P2 | Virgin's mantle | 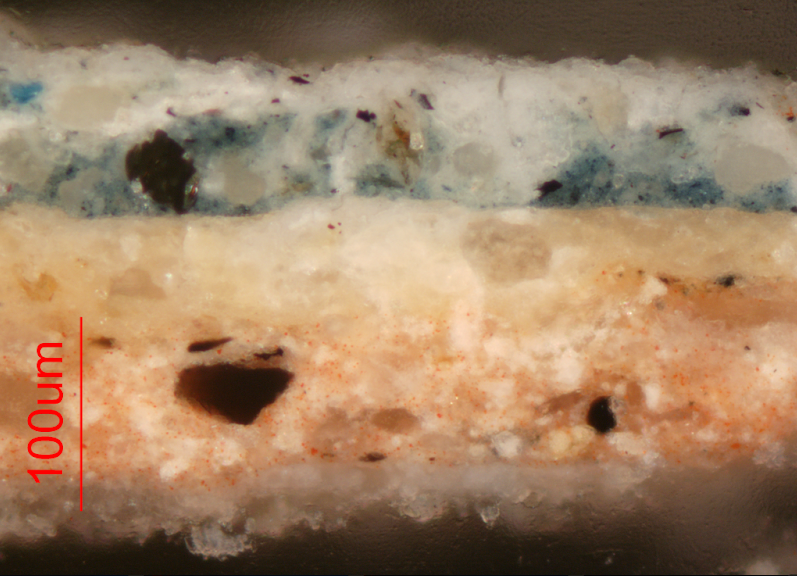  **1B**  **2**  **0** | EDXRF: **Pb**, Ca, Fe, [Cu], [K], [P], [Ti] | **2. Raman:**  1054 (vs)  116 (vs), 155 (s), 200 (s)  125 (s), 1051 (vs) | **Lead white (2PbCO_3_.Pb(OH)_2_ / PbCO_3_)**  Cotunnite PbCl_2_?  Plumbonacrite (Pb_10_(CO_3_)_6_O(OH)_6_)? |
|  |  |  |  | **1B. Raman:**  222 (vs), 291 (s), 405 (s), 608 (m), 654 (m), 1310 (m, br)  430 (w), 590 (vw), 960 (vs), 1070 (vw)  957 (m), 1328 (vs, br), 1575 (vs, br)  1053 (vs)  128 (m), 462 (s) | Haematite (α-Fe_2_O_3_)  Bone white (Ca_3_(PO_4_)_2_)  Bone black (C+Ca_3_(PO_4_)_2_)  Lead white (2PbCO_3_.Pb(OH)_2_ / PbCO_3_)  [Quartz (SiO_2_)] |
| P3 | Flesh paint | 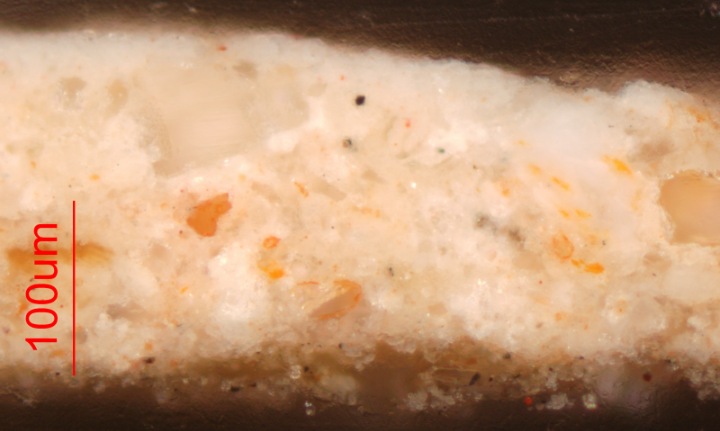  **2** |  | **2. Raman:**  206 (m), 461 (s)  152 (m), 280 (m), 711 (m), 1085 (vs)  174 (w), 299 (m), 1096 (vs)  102 (s), 1052 (vs)  223 (s), 243 (m), 291 (vs), 408 (vs), 492 (w), 608 (s), 656 (m, br)  118 (vs), 148 (m), 220 (w), 311 (w), 389 (w), 478 (vw), 547 (vs)  1325 (vs, br), 1595 (vs, br) | Quartz (SiO_2_)  Calcium carbonate (CaCO_3_)  Dolomite (CaMg(CO_3_)_2_)  Lead white (2PbCO_3_.Pb(OH)_2_ / PbCO_3_)  Haematite (α-Fe_2_O_3_)  [Red lead (Pb_3_O_4_)]  [Carbon black (C)] |
| P4 | Veil | 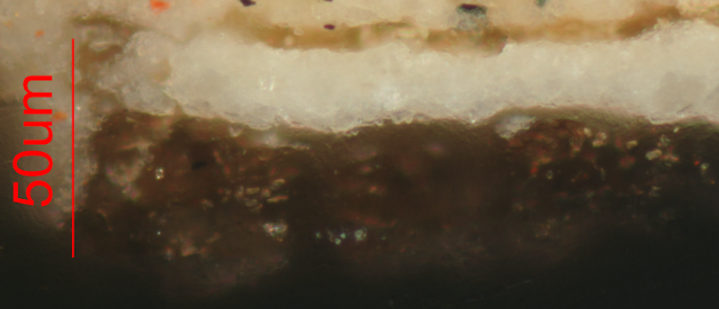  **1A**  **2** |  | **2. Raman:**  1053 (vs) | **Lead white (2PbCO_3_.Pb(OH)_2_ / PbCO_3_)** |
|  |  |  |  | **1A. Raman:**  121 (vs), 151 (m), 223 (w), 314 (w), 391 (w), 480 (vw), 550 (vs)  1049-1053 (vs, doublet)  1328 (vs, br), 1590 (vs, br)  253 (vs) | Red lead (Pb_3_O_4_)  Lead white (2PbCO_3_.Pb(OH)_2_ / PbCO_3_)  Carbon black (C)  Vermilion (HgS) |
| P5 | Virgin’s mantle | 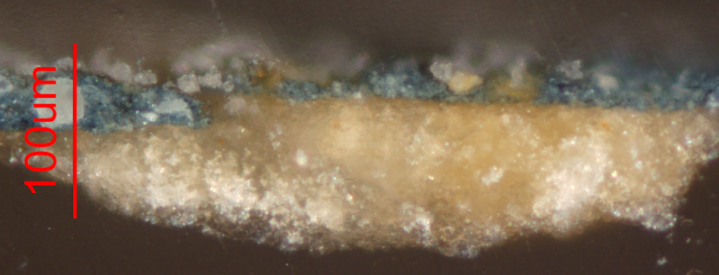  **0**  **2** | EDXRF: **Ca**, Pb, Fe, (Cu) | **2. Raman:**  102 (w), 829 (vw), 1054 (vs)  252 (m), 276 (w), 310 (vw), 546 (s), 600 (s), 675 (w), 1226 (vw), 1252 (w), 1574 (s), 1584 (s) | Lead white (2PbCO_3_.Pb(OH)_2_ / PbCO_3_)  Indigo (C_16_H_10_N_2_O_2_) |
| P6 | Virgin’s mantle | 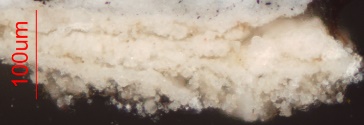  **2** |  | **2. Raman:**  104 (s), 1054 (vs)  110 (s), 125 (s), 1050 (vs) | **Lead white (2PbCO_3_.Pb(OH)_2_ / PbCO_3_)**  Plumbonacrite (Pb_10_(CO_3_)_6_O(OH)_6_)? |
| P7 | Virgin’s mantle | 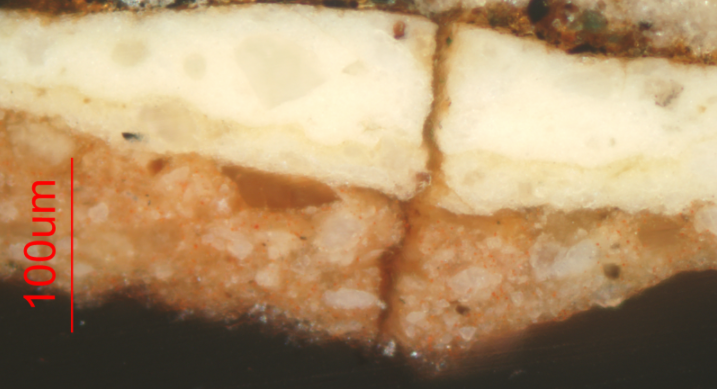  **2**  **1C**  **1B** |  | **2. Raman:**  104 (vs), 1054 (vs) | **Lead white (2PbCO_3_.Pb(OH)_2_ / PbCO_3_)** |
|  |  |  |  | **1C. Raman:**  1054 (vs) | **Lead white (2PbCO_3_.Pb(OH)_2_ / PbCO_3_)** |
|  |  |  |  | **1B. SEM-EDS:**  Pb, Ca, C, O, Cl, P, Si, K, Mg, Fe, Al  **Raman:**  104 (vs), 1054 (vs)  225 (vs), 295 (s), 410 (m), 610 (w, m), 1314 (m, br)  -  -  960 (m), 1327 (vs, br), 1596 (vs, br) | Lead white (2PbCO_3_.Pb(OH)_2_ / PbCO_3_)  Haematite (α-Fe_2_O_3_)  Calcium carbonate (CaCO_3_)  [Quartz (SiO_2_)]  [Bone black (C+Ca_3_(PO_4_)_2_)] |
| J1 | Angel’s vest | 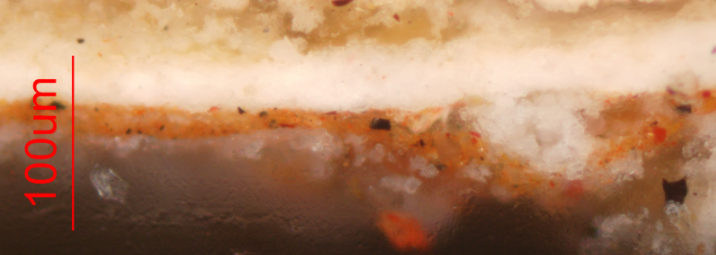  **1B**  **2** |  | **2. SEM-EDS:**  Pb, O, Cl  **Raman:**  1054 (vs)  221 (s), 295 vs), 408 (s), 608 (m), 656 (br, m)  1346 (vs, br), 1585 (vs, br)  1085 (vs)  108 (vs), 121 (s, sh), 324 (m)  118 (vs), 154 (s), 200 (s) | **Lead white (2PbCO_3_.Pb(OH)_2_ / PbCO_3_)**  [Haematite (α-Fe_2_O_3_)]  [Carbon black (C)]  [Calcium carbonate (CaCO_3_)]  [Laurionite Pb(OH)Cl]?  [Cotunnite PbCl_2_]? |
|  |  |  |  | **1B. SEM-EDS:**  O, Pb, Mg, Fe, Ca, Cl, Si, Ti, Al, K  **Raman:**  223 (s), 244 (m), 294 (vs), 408 (s), 610 (m), 657 (br, m)  250 (vs), 282 (w, sh), 341 (m)  130 (w), 153 (m), 200 (m), 289 (m), 308 (s), 352 (vs)  1332 (vs, br), 1593 (vs, br)  312 (w), 533 (w), 659 (vs) | **Haematite (α-Fe_2_O_3_)**  Vermilion (HgS)  Orpiment (As_2_S_3_)  Carbon black (C)  [Magnetite (Fe_3_O_4_)] |
| J2a | Loincloth | 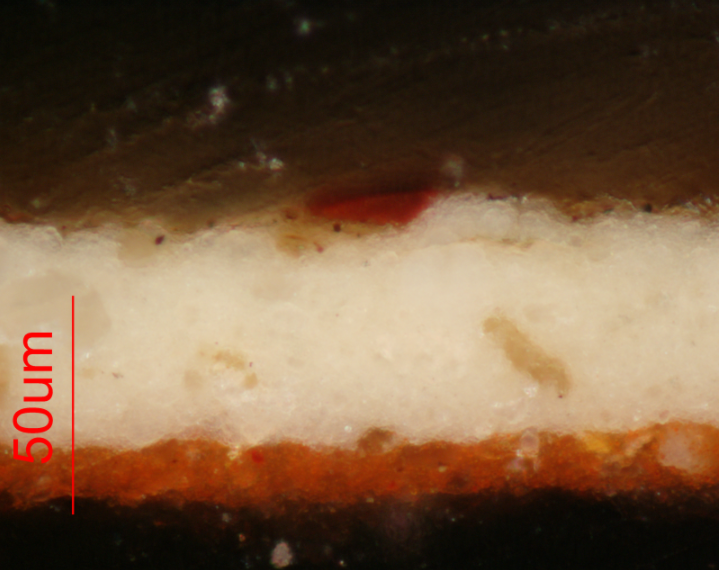  **1B**  **2** |  | **2. Raman:**  1053 (vs)  1085 (vs) | **Lead white (2PbCO_3_.Pb(OH)_2_ / PbCO_3_)**  Calcium carbonate (CaCO_3_) |
|  |  |  |  | **1B. Raman:**  225 (s), 294 (vs), 409 (s)  1087 (vs)  1054 (vs)  464 (vs)  142 (vs), 196 (w), 638 (w)  1325 (vs, br), 1594 (vs, br) | Haematite (α-Fe_2_O_3_)  Calcium carbonate (CaCO_3_)  Lead white (2PbCO_3_.Pb(OH)_2_ / PbCO_3_)  [Quartz (SiO_2_)]  [Anatase (TiO_2_)]  [Carbon black (C)] |
| J2b | Virgin’s mantle | 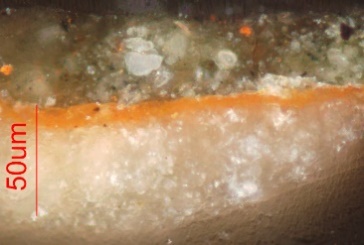  **3**  **1B**  **0**  **2** |  | **2. Raman:**  1049 (vs)  251 (w), 276 (vw), 545 (m), 598 (m), 674 (m), 1250 (s) ,1574 (s), 1585 (s)  252 (vs), 287 (w, sh), 341 (m)  1325 (vs, br), 1589 (vs, br)  1007 (vs) | Lead white (2PbCO_3_.Pb(OH)_2_ / PbCO_3_)  Indigo (C_16_H_10_N_2_O_2_)  Vermilion (HgS)  [Carbon black (C)]  [Calcium sulphate dihydrate (CaSO_4_.2H_2_O)] |
|  |  |  |  | **1B. Raman:**  225 (s), 294 (vs), 409 (s)  1087 (vs)  1054 (vs)  464 (vs)  142 (vs), 196 (w), 638 (w)  1325 (vs, br), 1594 (vs, br) | Haematite (α-Fe_2_O_3_)  Calcium carbonate (CaCO_3_)  Lead white (2PbCO_3_.Pb(OH)_2_ / PbCO_3_)  [Quartz (SiO_2_)]  [Anatase (TiO_2_)]  [Carbon black (C)] |
| J3 | Virgin’s dress | 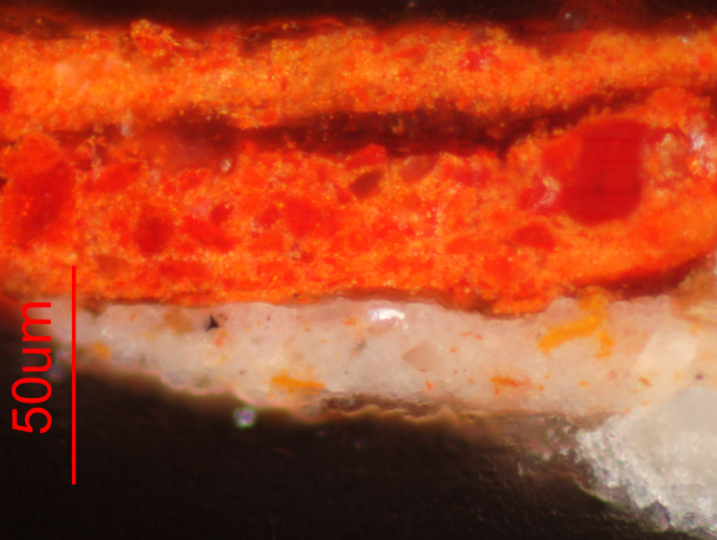  **2**  **0** |  | **3. Raman:**  252 (vs), 284 (sh, w), 342 (m) | Vermilion (HgS) |
|  |  |  |  | **2. Raman:**  1050-1052 (vs)  253 (vs), 285 (sh, w), 343 (m)  122 (vs), 153 (w), 223 (w), 391 (w), 551 (vs) | Lead white (2PbCO_3_.Pb(OH)_2_ / PbCO_3_)  Vermilion (HgS)  Red lead (Pb_3_O_4_) |
| J4 | Virgin’s mantle | 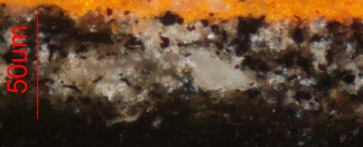  **2** |  | **2. Raman:**  1331 (vs, br), 1596 (vs, br)  1086 (vs)  242 (vw), 299 (w), 392 (vs), 475 (vw), 552 (w)  1008 (vs)  252 (vs), 342 (m) | **Carbon black (C)**  **Calcium carbonate (CaCO_3_)**  Goethite (α-FeOOH)  Calcium sulphate dihydrate (CaSO_4_.2H_2_O)  Vermilion (HgS) |
| J5a | Vest | 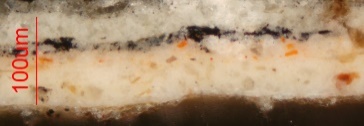  **2** |  | **2. Raman:**  681 (w), 837 (w), 1054 (vs)  126 (m), 462 (vs)  1325 (vs, br), 1591 (vs, br)  222 (vs), 290 (s), 407 (m), 607 (m), 660 (m) | **Lead white (2PbCO_3_.Pb(OH)_2_ / PbCO_3_)**  [Quartz (SiO_2_)]  [Carbon black (C)]  [Haematite (α-Fe_2_O_3_)] |
| J5b | Dalmatic | 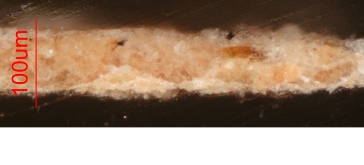  **2** | EDXRF: **Pb**, Ca, Ba, Fe, [Cu] | **2. Raman:**  1053 (vs)  226 (s), 244 (m), 293 (vs),411 (s), 616 (m)  1008 (vs)  1323 (vs, br), 1594 (vs, br)  252 (vs), 289 (w, sh), 343 (m) | Lead white (2PbCO_3_.Pb(OH)_2_ / PbCO_3_)  Haematite (α-Fe_2_O_3_)  Calcium sulphate dihydrate (CaSO_4_.2H_2_O)  [Carbon black (C)]  [Vermilion (HgS)] |
| J6 | Virgin’s mantle | 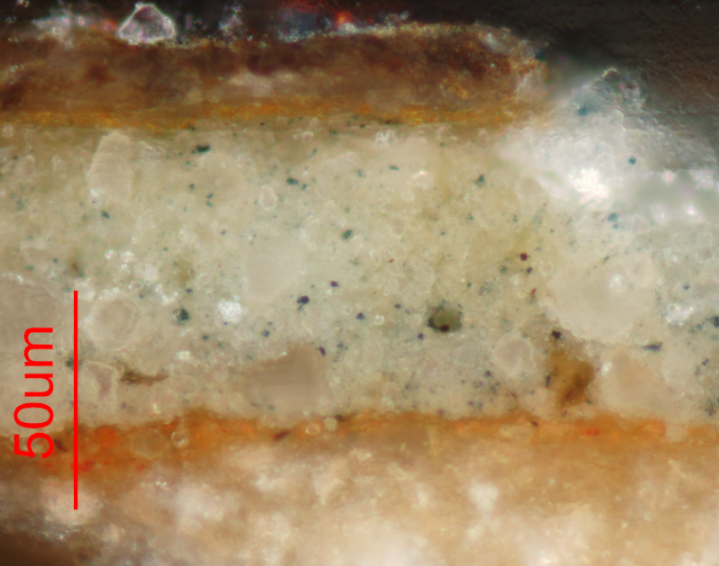  **1B**  **0**  **2** | EDXRF: **Pb**, Ca, Ba, Sn, Fe, Cu | **2. Raman:**  104 (w), 1055 (vs)  251 (m), 276 (w), 546 (s), 600 (s), 675 (w), 1228 (w), 1249 (m) | Lead white (2PbCO_3_.Pb(OH)_2_ / PbCO_3_)  Indigo (C_16_H_10_N_2_O_2_) |
|  |  |  |  | **1B. Raman:**  1054 (vs)  253 (vs), 284 (w, sh), 343 (m)  225 (vs), 293 (vs), 410 (s), 499 (br, w), 613 (br, w), 656 (br, w)  1321 (vs, br), 1607 (vs, br)  130 (vs), 196 (s)  141 (vs); 195 (vw); 393 (w); 515 (w); 637 (w) | Lead white (2PbCO_3_.Pb(OH)_2_ / PbCO_3_)  Vermilion (HgS)  Haematite (α-Fe_2_O_3_)  [Carbon black (C)]  [Lead tin yellow (type I) (Pb_2_SnO_4_)]  [Anatase (TiO_2_)] |
| J6 | Mantle lining | 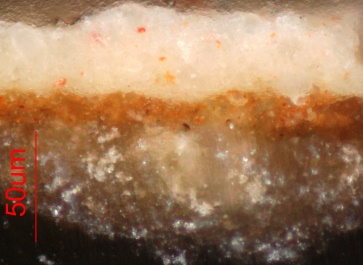  **2**  **0**  **1B** | EDXRF: **Pb**, Ca, Cu, Fe, Hg, [Ba] | **2. Raman:**  104 (w), 1054 (vs)  252 (vs), 282 (w, sh), 343 (m)  226 (vs), 295 (s), 413 (m), 501 (m), 615 (m), 663 (m), 1328 (m, br) | **Lead white (2PbCO_3_.Pb(OH)_2_ / PbCO_3_)**  Vermilion (HgS)  Haematite (α-Fe_2_O_3_) |
|  |  |  |  | **1B. Raman:**  1054 (vs)  253 (vs), 284 (w, sh), 343 (m)  225 (vs), 293 (vs), 410 (s), 499 (br, w), 613 (br, w), 656 (br, w)  1321 (vs, br), 1607 (vs, br)  130 (vs), 196 (s)  141 (vs); 195 (vw); 393 (w); 515 (w); 637 (w) | Lead white (2PbCO_3_.Pb(OH)_2_ / PbCO_3_)  Vermilion (HgS)  Haematite (α-Fe_2_O_3_)  [Carbon black (C)]  [Lead tin yellow (type I) (Pb_2_SnO_4_)]  [Anatase (TiO_2_)] |
| D1  **1B** | Shorts | 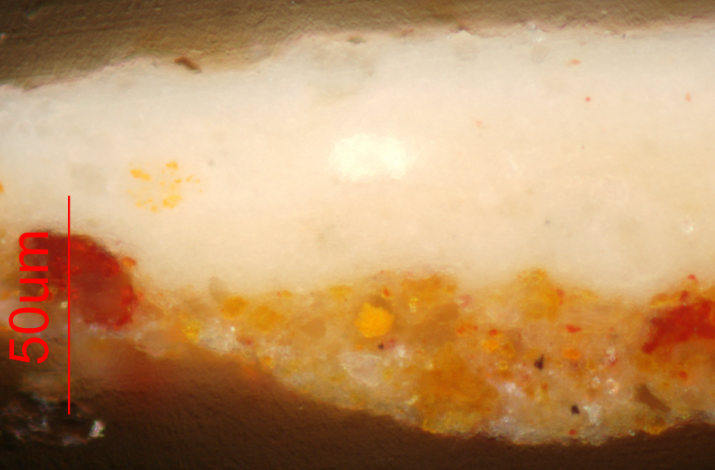  **1C**  **1B**  **2** | EDXRF: **Pb**, Fe, Cu, Ca | **2. SEM-EDS:**  Pb, C, O  **Raman:**  1054 (vs)  251 (vs), 280 (w, sh), 340 (m)  119 (vs), 150 (m), 220 (w), 314 (w), 389 (w), 479 (vw), 548 (vs) | **Lead white (2PbCO_3_.Pb(OH)_2_ / PbCO_3_)**  [Vermilion (HgS)]  [Red lead (Pb_3_O_4_)] |
|  |  |  |  | **1C. SEM-EDS:**  Pb, C, O, Ca  **Raman:**  1053 (vs)  119 (vs), 149 (m), 314 (w), 390 (w), 480 (vw), 548 (vs)  1325 (vs, br), 1602 (vs, br)  - | **Lead white (2PbCO_3_.Pb(OH)_2_ / PbCO_3_)**  [Red lead (Pb_3_O_4_)]  [Carbon black (C)]  [Calcium carbonate (CaCO_3_)] |
|  |  |  | EDXRF: **Ca**, **Pb**, Fe, Cu, [Sr], [Hg] | **1B. Raman:**  1054 (vs)  299 (m), 387 (vs)  223 (s), 243 (m), 291 (vs), 408 (s) 495 (w), 610 (m)  119 (vs), 149 (m), 222 (w), 311 (w), 337 (vw), 388 (w), 548 (vs)  252 (vs), 283 (w, sh), 343 (m)  1334 (vs, br), 1592 (vs, br)  158 (w), 1087 (vs)  127 (s), 1048, 1052, 1055 (vs) | Lead white (2PbCO_3_.Pb(OH)_2_ / PbCO_3_)  Goethite (α-FeOOH)  Haematite (α-Fe_2_O_3_)  Red lead (Pb_3_O_4_)  [Vermilion (HgS)]  [Carbon black (C)]  [Calcium carbonate (CaCO_3_)]  [Plumbonacrite (Pb_10_(CO_3_)_6_O(OH)_6_)]? |
| D2a | Virgin’s mantle | 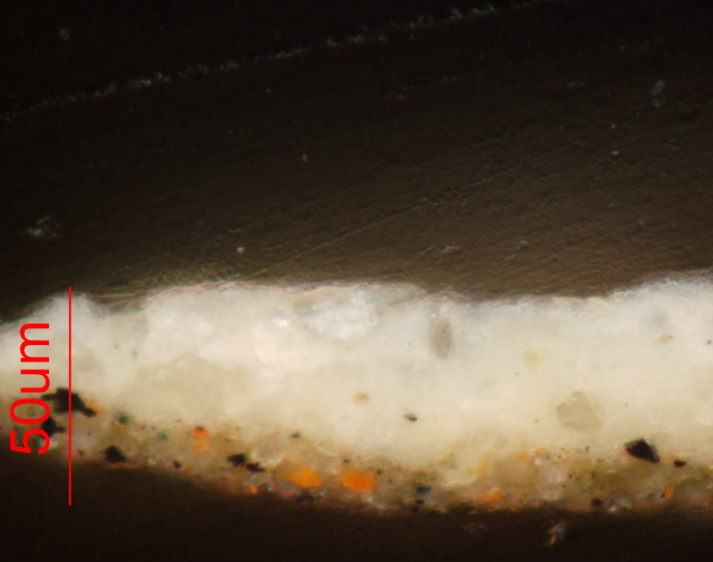  **2**  **1C**  **1B** |  | **2. SEM-EDS:**  Pb, C, O  Raman  1051 (vs)  1324 (vs, br), 1604 (vs, br) | **Lead white (2PbCO_3_.Pb(OH)_2_ / PbCO_3_)**  [Carbon black (C)] |
|  |  |  |  | **1C. Raman:**  1049-1052 (vs)  1340 (vs, br), 1592 (vs, br) | **Lead white (2PbCO_3_.Pb(OH)_2_ / PbCO_3_)**  [Carbon black (C)] |
|  |  |  |  | **1B. Raman:**  1049 (vs)  298 (s), 390 (s), 550 (w, br)  1325 (vs, br), 1594 (vs, br)  252 (vs), 285 (w, sh), 343 (m)  1085 (vs)  120 (vs), 149 (m), 221 (w), 313 (w), 389 (w), 479 (vw), 548 (vs)  127 (s), 1053 (vs) | Lead white (2PbCO_3_.Pb(OH)_2_ / PbCO_3_)  Goethite (α-FeOOH)  Carbon black (C)  Vermilion (HgS)  Calcium carbonate (CaCO_3_)  Red lead (Pb_3_O_4_)  [Plumbonacrite (Pb_10_(CO_3_)_6_O(OH)_6_)]? |
| D2b | Flower | 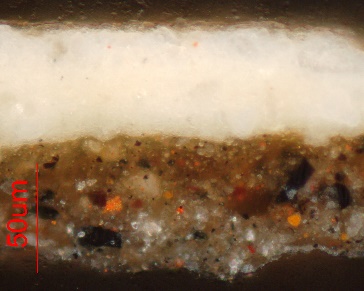  **2**  **1B**  **1C** | EDXRF: **Pb**, Cu, [Ca], [Fe], [Hg] | **2. Raman:**  103 (m), 1056 (vs)  254 (vs), 286 (w, sh), 344 (m) | **Lead white (2PbCO_3_.Pb(OH)_2_ / PbCO_3_)**  [Vermilion (HgS)] |
|  |  |  |  | **1C. Raman:**  1049 (vs)  1322 (vs, br), 1593 (vs, br) | Lead white (2PbCO_3_.Pb(OH)_2_ / PbCO_3_)  [Carbon black (C)] |
|  |  |  |  | **1B. Raman:**  1049 (vs)  298 (s), 390 (s), 550 (w, br)  1325 (vs, br), 1594 (vs, br)  252 (vs), 285 (w, sh), 343 (m)  1085 (vs)  120 (vs), 149 (m), 221 (w), 313 (w), 389 (w), 479 (vw), 548 (vs)  127 (s), 1053 (vs) | Lead white (2PbCO_3_.Pb(OH)_2_ / PbCO_3_)  Goethite (α-FeOOH)  Carbon black (C)  Vermilion (HgS)  Calcium carbonate (CaCO_3_)  Red lead (Pb_3_O_4_)  [Plumbonacrite (Pb_10_(CO_3_)_6_O(OH)_6_)]? |
| D3 | Flesh paint | 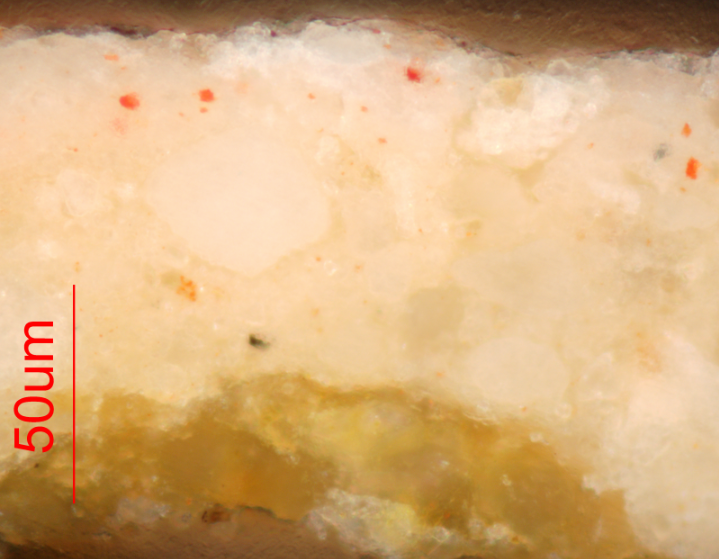  **1B**  **2**  **3** | EDXRF: **Pb**, Fe, Ca, [Cu], [Hg], [K] | **3. Raman:**  1052 (vs)  249 (vs), 281 (w, sh), 339 (m) | **Lead white (2PbCO_3_.Pb(OH)_2_ / PbCO_3_)**  Vermilion (HgS) |
|  |  |  |  | **2. Raman:**  1052 (vs)  1084 (vs)  299 (m), 386 (vs)  220 (vs), 289 (s), 403 (s)  1334 (vs, br), 1579 (vs, br)  126 (w), 1048 (vs) | Lead white (2PbCO_3_.Pb(OH)_2_ / PbCO_3_)  Calcium carbonate (CaCO_3_)  Goethite (α-FeOOH)  [Haematite (α-Fe_2_O_3_)]  [Carbon black (C)]  [Plumbonacrite (Pb_10_(CO_3_)_6_O(OH)_6_)]? |
|  |  |  |  | **1B. Raman:**  224 (vw), 299 (m), 390 (vs), 550 (m)  153 (vw), 279 (vw), 711 (w), 1085 (vs)  463 (vs) | Goethite (α-FeOOH)  Calcium carbonate (CaCO_3_)  Quartz (SiO_2_) |


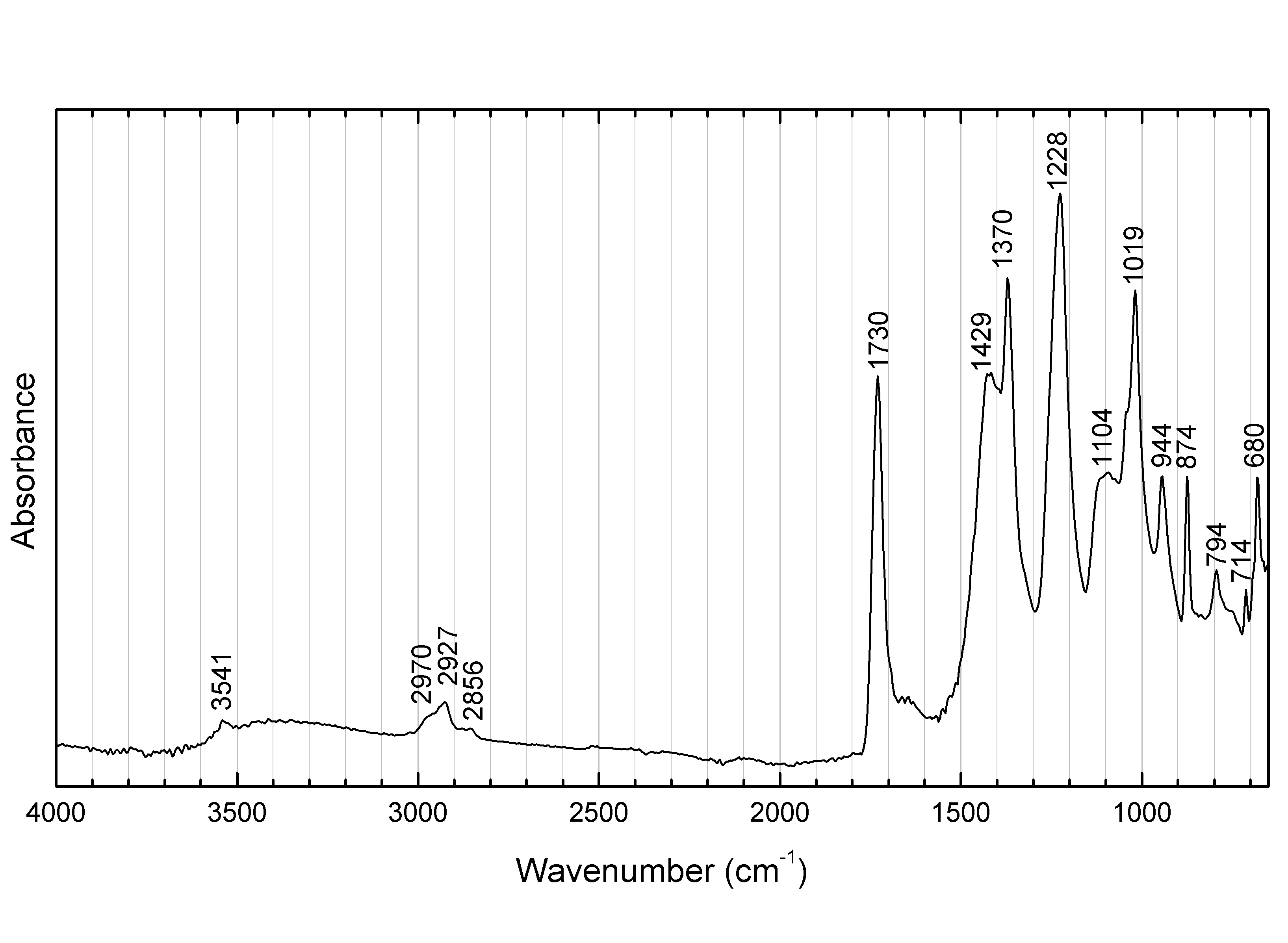


Figure S1. FTIR-ATR spectrum from the surface of a sample collected near the sampling area of P1 identified PVAc, a common consolidant material. An intense band was observed at 1730 cm^-1^, corresponding to the C=O stretching vibration of the acetate group, followed by two bands at 1429 and 1370 cm^-1^, which correspond to the CH_2_ and CH_3_ bending vibrations. A very intense band identified at 1228 cm^-1^ is attributed to the asymmetric stretching mode of ester groups and is followed by a band at 1019 cm^-1^ attributed to the vibrations of the C-C group. The less intense bands identified at 944 and 794 cm^-1^ are attributed to rocking vibrations of CH_3_ and CH_2_. Additionally, weak absorption bands at 2970, 2927 cm^-1^ correspondent to CH_3_ and CH_2_ asymmetric stretching vibrations, and a band at 2856 cm^-1^ were also identified^50,51^.


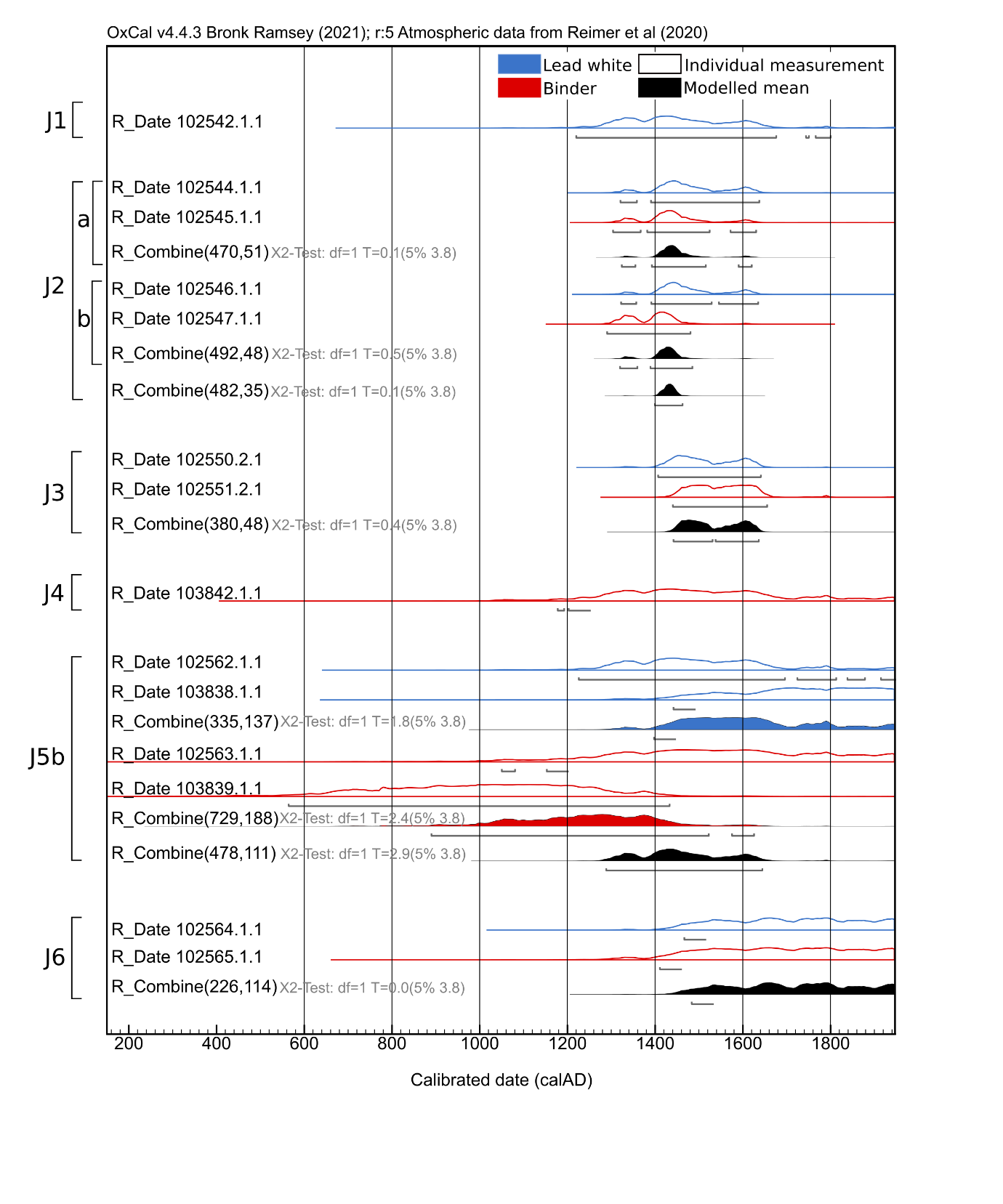


Figure S2. Calibrated ^14^C ages for the samples of João Afonso’s workshop. The simple radiocarbon calibration of the ^14^C ages of lead white (blue) and binder (red) are displayed as outlines while the solid distributions represent their mean value using the combine function in Oxcal.


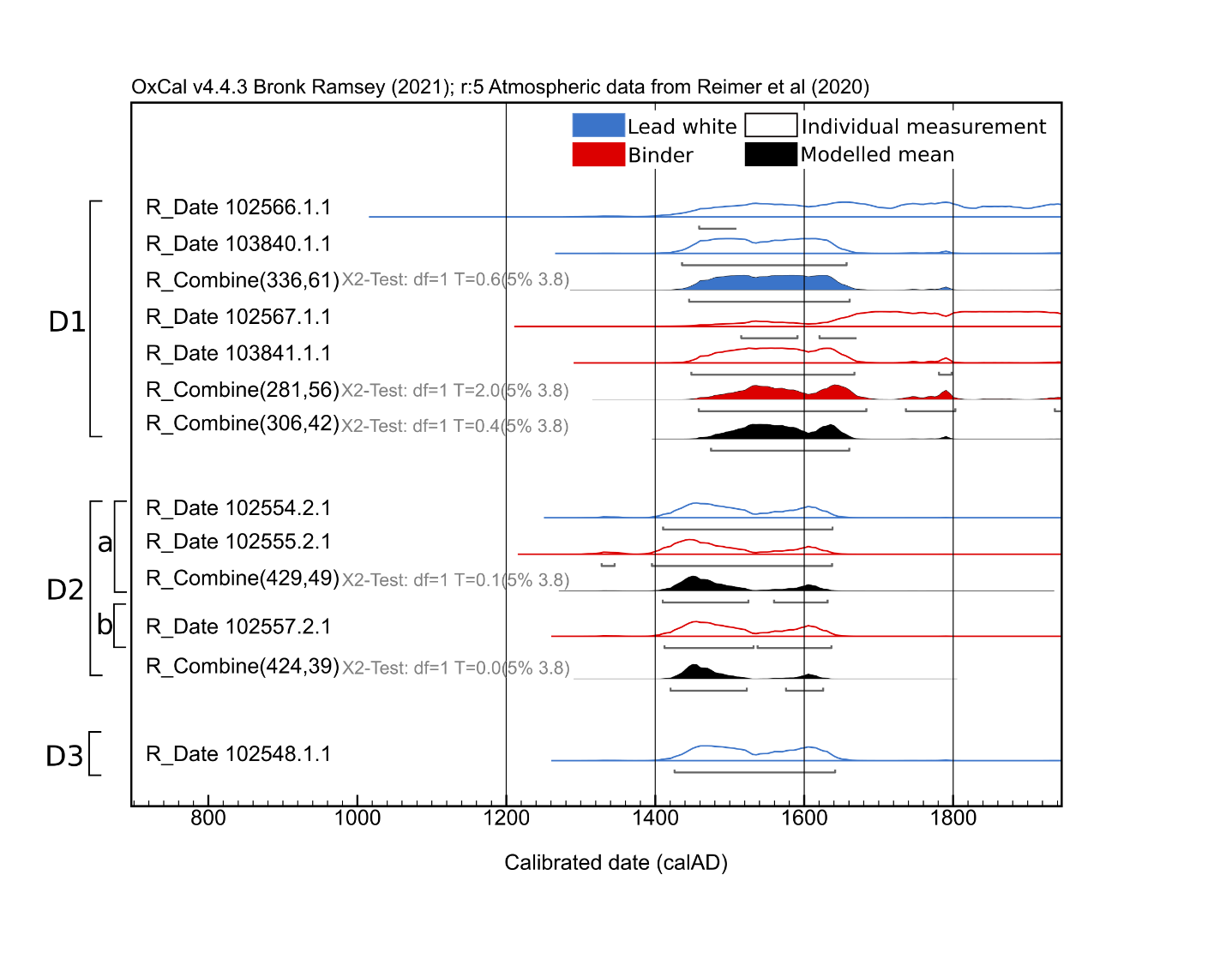


Figure S3. Calibrated ^14^C ages for the samples of Diogo Pires-o-Velho’s workshop. The simple radiocarbon calibration of the ^14^C ages of lead white (blue) and binder (red) are displayed as outlines while the solid distributions represent their mean value using the combine function in Oxcal.


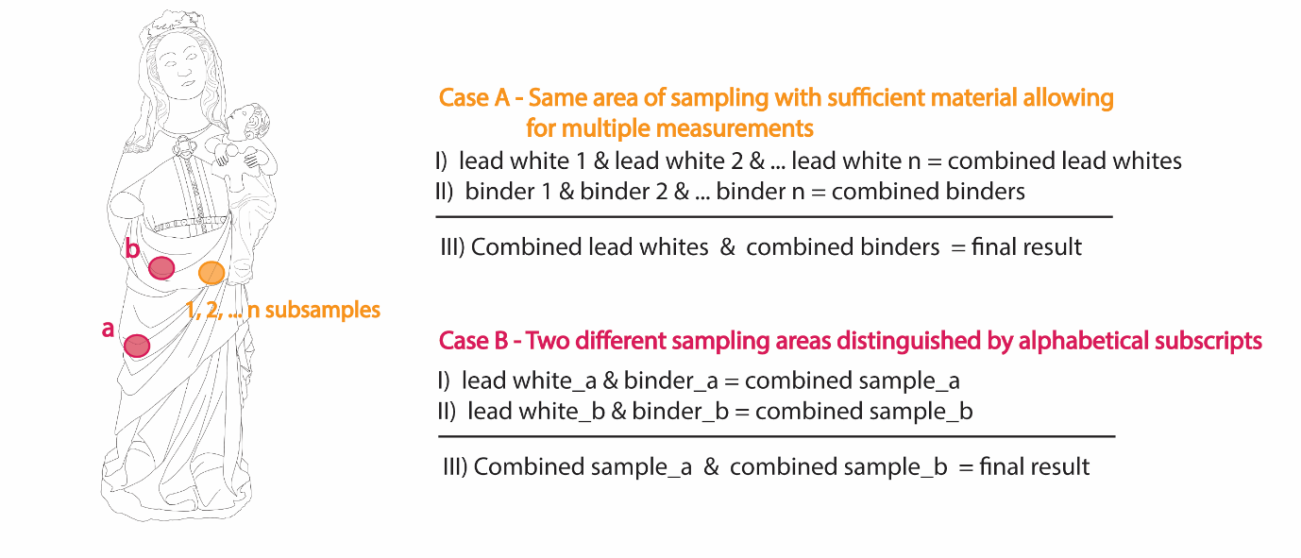


Figure S4. Scheme illustrating the two cases (A and B) of sampling and the strategy adopted for the modelled mean in each case.


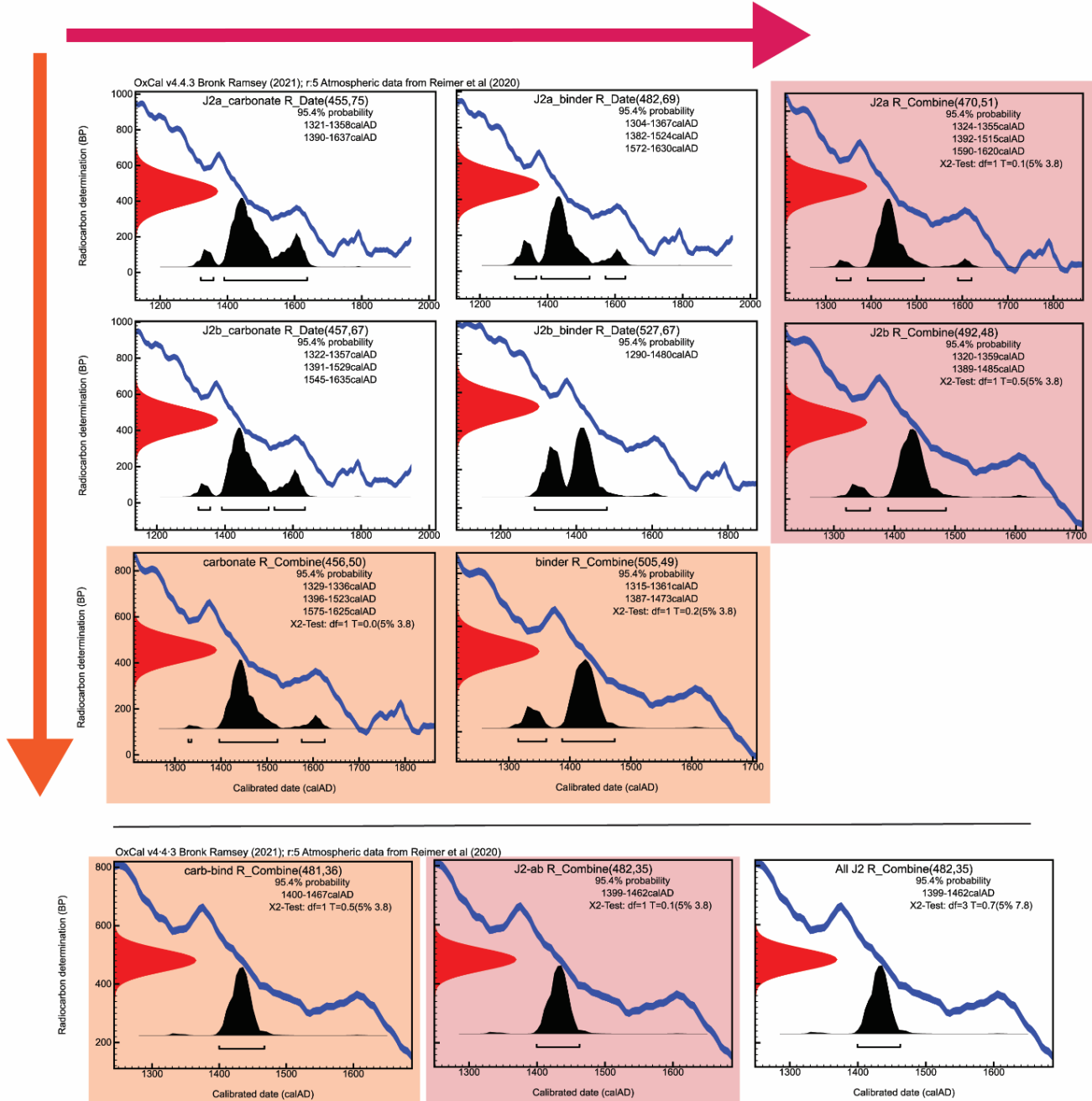


Figure S5. The combination of multiple ^14^C measurements is justifiable if they relate to the same event. Here the question is whether one combines the ^14^C dates from the individual sampling sites (horizontal) or if the pooling of dates should be done on the material, the lead white pigment or natural binder respectively (vertical). Sample location and sampling was conducted following material characterization studies, however series of superimposed repaints render the sampling difficult and ensuring that the same paint event is being sampled in two different areas is not always possible. Here we observe a perfect agreement of the two sampling sites, regardless on how the data is combined. Treating the data individually per location allows to confirm that sample J2a and J2b belong to the same polychromy campaign (validated by the chi-square test T=0.1% (5% 3.8) in the pink shaded lower figure). When considering that the material used in both locations, the chi-square confirms that it is the same lead white, respectively same binder material which was admixed and used in both location (lower orange shaded plot), thus confirming the stratigraphy. The final three plots – the combination of the materials, the two sampling sites and simply all four ^14^C dates directly – show that there is no difference in the final calibrated results.
